# GABAergic and α-Glucosidase-Inhibitory Potentials of Fractions and Isolated Xanthones from *Hypericum revolutum* Vahl subsp. *revolutum*

**DOI:** 10.3390/molecules30173530

**Published:** 2025-08-29

**Authors:** Maria S. Chukwuma, Lorenza Bertaina, Sophia Khom, Chika I. Chukwuma, Pieter C. Zietsman, Anke Wilhelm, Susanna L. Bonnet

**Affiliations:** 1 Department of Chemistry, Faculty of Natural and Agricultural Sciences, University of the Free State, Bloemfontein 9301, South Africa; 2017373891@ufs4life.ac.za (M.S.C.); wilhelma@ufs.ac.za (A.W.); 2 Department of Pharmaceutical Sciences, Faculty of Life Sciences, University of Vienna, 1090 Vienna, Austria; lorenza.bertaina@univie.ac.at (L.B.); sophia.khom@univie.ac.at (S.K.); 3 Vienna Doctoral School of Pharmaceutical, Nutritional and Sport Sciences (PhaNuSpo), University of Vienna, 1090 Vienna, Austria; 4 Center for Quality of Health and Living (CQHL), Faculty of Health and Environmental Sciences, Central University of Technology, Bloemfontein 9301, South Africa; cchukwuma@cut.ac.za; 5 Bloemfontein National Museum, Bloemfontein 9301, South Africa; zietspc@gmail.com

**Keywords:** *H. revolutum*, xanthones, GABA_A_ receptor, I_GABA_ modulation, α-glucosidase inhibition

## Abstract

This study aimed to investigate the glycaemic control potential and modulation of GABA-induced chloride currents (I_GABA_) of *H. revolutum* and the possible bioactive xanthones. Fractions from the leaf and stem extracts (dichloromethane and methanol) were assessed for in vitro α-glucosidase-inhibitory potential and their ability to modulate I_GABA_ (GABAergic effect) through GABA_A_ receptors heterologously expressed in *Xenopus* oocytes. Xanthones 4-hydroxy-2,3-dimethoxy-9*H*-xanthen-9-one (**1**), 3-hydroxy-2,4-dimethoxy-9*H*-xanthen-9-one (**2**) and *trans*-3-(4-hydroxy-3-methoxyphenyl)-2-(hydroxymethyl)-5-methoxy-2,3-dihydro-*7H*-[1,4]dioxino[2,3-c]xanthen-7-one (**3**) were isolated from the stem and tested in the GABA_A_ receptors assay, but only **3** was assessed for α-glucosidase-inhibitory action. Compared to acarbose (IC_50_ = 6.16 µM), **3** showed a mild to moderate α-glucosidase-inhibitory activity (IC_50_ = 45.1 µM), which may be attributed to the absence of a hydroxyl group at its xanthone core. Isomeric compounds **1** and **2** significantly enhanced I_GABA_ with similar efficacy, while **3** was inactive, which may be attributed to its notable structural difference (cyclic ether substitution) compared to compounds **1** and **2**. *H. revolutum* stem contains xanthones with α-glucosidase-inhibitory potential, which also enhance I_GABA_ and could be further studied as a medicinal plant for managing GABA_A_ receptor-mediated mental disorders and/or diabetes.

## 1. Introduction

Comorbid mental depression and diabetes are significant clinical challenges, and depression is also widespread in people with diabetes [1,2]. Diabetes comprises a range of metabolic disorders, with abnormally high blood glucose being the most prominent clinical diagnostic marker [3]. The metabolic disorders of diabetes include impaired nutrient metabolism in many tissues, particularly in peripheral tissues, which collectively lead to poor circulating glucose homeostasis [3]. Inhibitors of carbohydrate digestive enzymes (glucosidase and amylase) are known therapeutic agents for diabetes because they suppress postprandial blood glucose increase [4].

Diabetic patients have an increased risk of having mental health issues like depression and anxiety [5]. In South Africa, anxiety, mood, depression, and drug use disorders are known mental illness that has a 12-month prevalence estimate of 16.5%, with 30.3% of the population reported to have suffered from a known mental disorder in their lifetime. The percentages are increasing compared to the global prevalence estimates from the World Health Organisation (WHO) World Mental Health surveys [6].

Gamma-aminobutyric acid (GABA) is an amino acid that is a primary inhibitory neurotransmitter in the brain [7]. GABA is released into the synaptic cleft and binds to postsynaptic GABA_A_ receptors. Binding to the receptor results in the opening of the integral chloride channel, which enables chloride influx to hyperpolarise the neuron, thereby inhibiting the generation and propagation of action potential [7]. Dysregulated GABA_A_ receptor signalling has been linked to a variety of neurologic and psychiatric disorders, including insomnia, anxiety, depression or epilepsy [8,9]. Synthetic and clinically applied GABA_A_ receptor modulators such as benzodiazepines, non-benzodiazepines (such as zolpidem), neuroactive steroids or even barbiturates still belong to the most important and widely used drugs.

Despite the pharmacological benefits of synthetic drugs modulating GABA_A_ receptors and antihyperglycaemic drugs, they are often associated with several unpleasant side effects. For example, the application of benzodiazepines is associated with multiple unwanted effects, including a high potential for tolerance, dependence, and abuse [10], while the gastrointestinal adverse effects of many old and new synthetic antidiabetics have been documented [11]. It is, therefore, imperative to search for alternative and/or complementary therapies. Plants are known to contain polyphenols, which possess medicinal potential. Their medicinal prospects in several diseases cannot be overemphasised [12]. Moreover, the potential use of medicinal plants for anxiety, depression, or stress treatment has been documented [13], while many plants have been reported to demonstrate antidiabetic potential through different mechanisms [14,15].

*Hypericum* is a vast plant genus of herbs or shrubs [16], containing about 500 species [17]. They are commonly used traditionally to treat depression or mental illness and have been used as an astringent, antipyretic, diuretic, antiphlogistic, analgesic, antidiabetic, and antidepressant in Europe, America, Africa, and Asia [18]. Several xanthones, including 1-(5,7-dihydroxy-2,2,6-trimethyl-2H-chromen-8-yl)-2-methylpropan-1-one, 8-isobutyryl-2,2,6-trimethyl-2H-chromene-5,7-diyl diacetate, 1,1′-(5,8,10-trihydroxy-2,2,6,9,13,13-hexamethyl-1,7a,13a,13b-tetrahydro-2*H*,13*H*-pyrano[3,2-c:4,5,6-d′e′]dichromene-4,11-diyl)bis(2-methylpropan-1-one) [19], hyperevolutin A, hyperevolutin B [20], and hyperevolutin C [21], have been reported in species of the *Hypericum* genus. In South Africa, *Hypericum perforatum* is among the common psychotropic plants which are used to treat depression [22,23]. An earlier in vitro study showed that *H. perforatum* inhibits the effects of serotonin, noradrenaline, GABA, and L-glutamate [22,24].

*Hypericum revolutum* Vahl subsp. *revolutum* (Hypericaceae) is a related species. It is a multi-stemmed shrub popular throughout sub-Saharan Africa [25]. It is a native of South Africa and Zimbabwe, but found in Southwest Arabia, Fernando, the Comoro Islands, the Indian Ocean Islands, and Cameroon. In South Africa, the plant grows in the Gauteng, Eastern Cape, Mpumalanga, KwaZulu-Natal provinces, and the Cape coastal area [26].

Medicinally, it is traditionally used to treat stomach aches and rheumatism. In Uganda, herbalists use it to treat tuberculosis, while in Cameroon, it is used to treat malaria and other illnesses such as skin infection, tumours, infertility, and, notably, epilepsy [26,27,28,29]. Pharmacological studies have shown the antifungal [19], antidepressant [30,31], anti-inflammatory [32], antimicrobial [33], and colonic anticancer [20] properties of *H. revolutum*. However, knowledge about its GABAergic effects and glycaemic control potential remains elusive. Hence, the present study undertook the fractionation and isolation of potential, novel GABA_A_ receptor modulators and α-glucosidase-inhibitory compounds from *H. revolutum* stem and leaves.

## 2. Results and Discussion

### 2.1. Yield and Qualitative Phytochemical Analyses

The percentage yield of the MeOH (after tannin removal) and DCM extracts of the PS was 8.4% and 1.5%, respectively. In comparison, the percentage of the MeOH (after tannin removal) and DCM extracts of the PL were 2.5% and 3.5%, respectively. The qualitative phytochemical profile of the PS extracts is shown in Table S1 of the Supplementary Data. The MeOH extract showed a greater abundance of phenolics and flavonoids than the DCM extract because it is a more polar solvent [34].

### 2.2. Fractions and Isolated Compounds

**Figure 1:**
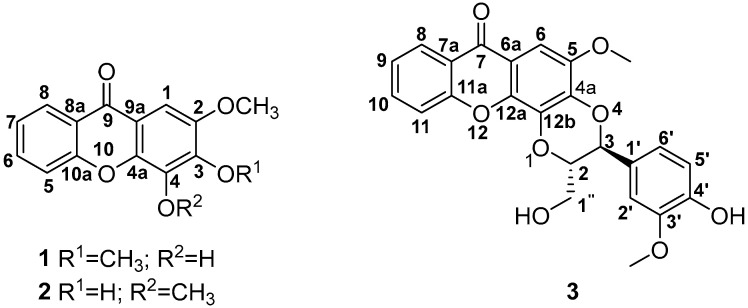
Structures of the isolated compounds **1**, **2** and **3**.

The solvent systems and yields of fractionation and/or sub-fractionation are presented in the Supplementary Data (Tables S2–S6). Fourteen fractions were obtained from the DCM extract of the PS, while twenty-six fractions were obtained from the MeOH extract of the PS. The PL afforded eleven and four fractions from the DCM and MeOH extracts, respectively. Compound **1** (3.9 mg) (Figure 1) was isolated from combined fractions F8 and F9 (HEX:EtOAc; 7:3 *v*/*v*) obtained from the DCM extract of the PS using a preparative TLC plate (HEX:Acetone; 8:2 *v*/*v*) (Figure S1). Compound **3** (14.8 mg) (Figure 1) was isolated from fraction F12 (CHCl_3_:MeOH; 1:1 *v*/*v*) obtained from the DCM extract of the PS using a preparative TLC plate (HEX: EtOAc; 5:5 *v*/*v*) (Figure S2). The schematic diagram showing the isolation steps of compounds **1** and **3** is shown in Figure S3. Fraction F18 (CHCl_3_:MeOH; 9.5:0.5 *v*/*v*) from the MeOH extract of the PS yielded ten sub-fractions (Figure S4). Sub-fractions 6 and 7 were combined. Compound **2** (4.7 mg) (Figure 1) was isolated from the combined sub-fractions using the same solvent system (CHCl_3_:MeOH; 9.5:0.5 *v*/*v*) (Figure S4). The schematic diagram showing the isolation steps of compound **2** is shown in Figure S5. NMR spectroscopy, mass spectrometry (MS), infrared spectroscopy (IR), and literature comparisons [35] revealed that compounds **1**, **2**, and **3** are 4-hydroxy-2,3-dimethoxy-9*H*-xanthen-9-one, 3-hydroxy-2,4-dimethoxy-9*H*-xanthen-9-one, and *trans*-kielcorin, respectively. All three compounds have been isolated before, and *trans*-kielcorin has been reported from several *Hypericum* species [35]. However, this is the first time compounds **1**, **2**, and **3** were isolated from *H. revolutum*.

#### 2.2.1. Compound **1** (4-Hydroxy-2,3-dimethoxy-9*H*-xanthen-9-one)

The HR-ESIMS spectrum of compound **1** (Plate S1) showed an [M+H]^+^ pseudomolecular ion peak at *m*/*z* 273.0765, which corresponds to the molecular formula of C_15_H_12_O_5_ (calc. (M+H)^+^ *m*/*z* 273.0763). The IR spectrum (Plate S2) showed the OH bond stretching vibration at 3250 cm^−1^. At 2998, 2920, and 2851 cm^−1^, the C-H symmetrical and asymmetrical stretching vibrations were observed as intense, sharp peaks. Double bonds exhibited absorption at 1641 cm^−1^, C-O vibration at 1036 cm^−1^, and C-OH bond absorption at 1095 cm^−1^. The peak centred at 1731 cm^−1^ was determined to be C=O (ketone) stretching vibrations.

The ^1^H NMR spectrum (Plate S3) showed 12 proton resonances; four aromatic hydrogens in an AMPX system at δ_H_ 8.35 (dd, *J* = 8.1, 1.7 Hz, 1H, H-8), 7.72 (ddd, *J* = 8.5, 7.1, 1.7 Hz, 1H, H-6), 7.60 (dd, *J* = 8.5, 1.1 Hz, 1H, H-5), and 7.39 (ddd, *J =* 8.1, 7.1, 1.1 Hz, 1H, H-7), an aromatic singlet at δ_H_ 7.36 (s, 1H, H-1), a broadened singlet at δ_H_ 6.06 (br s, OH, 1H), and two methoxy singlets at δ_H_ 4.08 (s, 3H) and 3.99 (s, 3H), respectively (Table S7).

**Figure 2:**
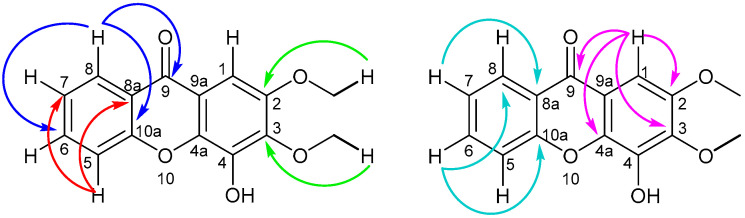
Two-dimensional HMBC correlations of H-1, H-5, H-6, H-7, H-8, and the methoxy hydrogens of compound **1**.

The ^13^C NMR (Plate S4) of **1** displayed 15 resonances: 13 aromatic carbons, of which three were oxygenated, and two oxygenated aliphatic carbons (Table S7). A comparison of the ^13^C NMR and ^13^C APT spectra (Plate S5) indicated five methine carbons, two methoxy carbons, a carbonyl carbon, and seven quaternary carbons. The 2D HSQC (Plate S7) and HMBC (Plate S8) spectra defined the direct correlations and long-distance correlations between the carbons and the hydrogens, respectively (Table S7). Long-distance correlations between H-8 (δ_H_ 8.35) with C-6 (δ_C_ 134.3), C-9 (δ_C_ 176.4), and C-10a (δ_C_ 155.9) were observed (Figure 2). H-5 (δ_H_ 7.60) correlated with C-8a (δ_C_ 121.3) and C-7 (δ_C_ 123.9), respectively, and H-6 (δ_H_ 7.72) correlated with C-10a and C-8, respectively. H-7 (δ_H_ 7.39) showed a correlation with C-8a (δ_C_ 121.3), and H-1 (δ_H_ 7.36) with C-9 (δ_C_ 176.4), C-4a (δ_C_ 137.9), C-2 (δ_C_ 149.4) (weak correlation), and C-3 (δ_C_ 141.1) (strong correlation). However, our assignments of C-2 and C-3 differ from those reported by Abdallah et al. [21] but are similar to those of Castelão et al. [36] (Table S7). Also, the HMBC correlation of the OC**H**_3_ (δ_H_ 3.98) with C-2 (δ_C_ 149.4) and OC**H**_3_ (δ_H_ 4.08) with C-3 (δ_C_ 141.1) confirmed the proton assignments.

Compound **1** was identified as 4-hydroxy-2,3-dimethoxy-9*H*-xanthan-9-one, and all physical data corresponded to published data [36].

#### 2.2.2. Compound **2** (3-Hydroxy-2,4-dimethoxy-9*H*-xanthen-9-one)

Compound **2** is a regio-isomer of **1** and was isolated as a yellow amorphous powder (R_f_ 0.16, CHCl_3_: MeOH, 9.5:0.5). The HR-ESIMS spectrum (Plate S9) showed an [M+H]^+^ pseudomolecular ion peak at *m*/*z* 273.0759, which corresponds to the molecular formula of C_15_H_12_O_5_ (calc. (M+H)^+^ *m*/*z* 273.0763). The IR spectrum (Plate S10) showed a broad peak at 3379 cm^−1^ corresponding to an OH bond stretching vibration. Peaks at 2924 and 2853 cm^−1^ were confirmed as the C-H symmetrical and asymmetrical stretching vibrations, while peaks at 1644 cm^−1^ indicated double bonds. The peak centering at 1727 cm^−1^ indicates the C=O (ketone) stretching vibration.

The ^1^H NMR spectrum of **2** (Plate S11) is almost identical to that of **1**, showing the same aromatic AMPX and ABX systems (Table S8). The singlet aromatic hydrogen is observed at δ_H_ 7.53 (s, 1H, H-1), the OH group as a broad resonance at δ_H_ 6.27, and two methoxy singlets at δ_H_ 4.13 (s, 3H) and 4.03 (s, 3H). The only significant difference is the observed downfield shifts of H-1 [δ_H_ 7.53 (**2**) and 7.36 (**1**)], the methoxy resonances (δ_H_ 4.13 and 4.03, respectively, for **2**, and 4.08 and 3.98, respectively, for **1**), and C-4a (δ_c_ 146.1 (**2**) and 137.9 (**1**)) (Tables S6 and S7). This indicated that the substitution pattern on the second aromatic ring of **2** differs from that of **1**. The correlations in the 2D COSY spectrum of **2** (Plate S14) are almost identical to those of **1** (Plate S6) since **2** displays the same spin systems as **1**.

**Figure 3:**
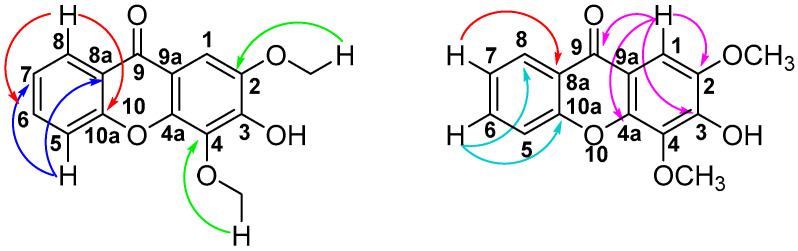
Two-dimensional HMBC correlations of H-1, H-5, H-6, H-7, H-8, and the methoxy hydrogens of compound **2**.

The ^13^C NMR (Plate S12) and ^13^C APT spectra (Plate S13) displayed 15 carbon signals: five methine carbons, two methoxy carbons, a carbonyl carbon, and seven quaternary carbons. The significant correlations observed in the 2D HMBC spectrum (Plate S16) are illustrated in Figure 3. Correlations of the aromatic ring on the left were similar to those observed for **1** and confirmed all assignments. H-1 at δ_H_ 7.53 correlated with C-9, C-4a, C-2, and C-3 at δ_C_ 176.1, 146.1, 145.0, and 144.8, respectively. The methoxy protons at δ_H_ 4.13 correlate with C-2, and δ_H_ 4.03 with C-4.

Comparison of the physical data with the previously published data [36] identified **2** as 3-hydroxy-2,4-dimethoxy-9*H*-xanthen-9-one.

#### 2.2.3. Compound **3** [*trans*-3-(4-Hydroxy-3-methoxyphenyl)-2-(hydroxymethyl)-5-methoxy-2,3-dihydro-*7H*-[1,4]dioxino[2,3-c]xanthen-7-one or kielcorin)]

**Figure 4:**
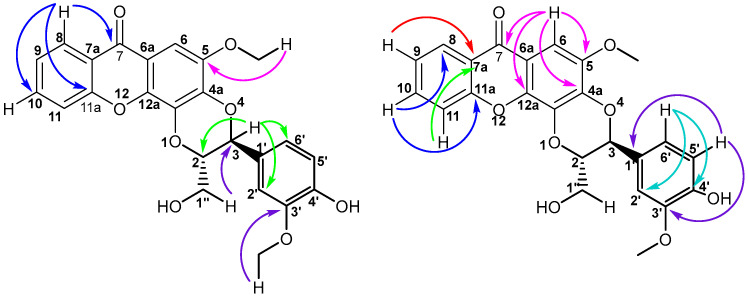
Two-dimensional HMBC correlations of compound **3**.

Compound **3** (Figure 1) was isolated as a yellow amorphous powder from the DCM extract of the plant stem (R_f_ 0.25, Hx:EtOAc, 5:5). The HR-ESIMS spectrum in negative mode (Plate S17) showed an [M-H]^−^ pseudomolecular ion peak at *m*/*z* 435.1061, which corresponds to the molecular formula of C_24_H_20_O_8_ (calc. (M-H)^−^ *m*/*z* 435.1080). The IR spectrum (Plate S18) displayed peaks at 3426 cm^−1^ (OH bond stretching vibration), 2921 and 2851 cm^−1^ (C-H symmetrical and asymmetrical stretching vibrations), 1607 cm^−1^ (double bonds), 1062 cm^−1^ (C-O absorption), 1081 cm^−1^ (C-OH), and 1726 cm^−1^ (carbonyl absorption). The ^1^H NMR spectrum of **3** (Plate S19) showed 20 proton resonances (Table S9). The aromatic region displayed eight aromatic hydrogens, which included one AMPX system with resonances at δ_H_ 8.39 (dd, *J* = 8.0, 1.7 Hz, 1H, H-8), 7.74 (td, *J* = 8.5, 7.0, 1.7 Hz, 1H, H-10), 7.61 (dd, *J* = 8.5, 1.0 Hz, 1H, H-11), and 7.43 (td, *J* = 8.0, 7.0, 1.0 Hz, 1H, H-9), and an ABX system at δ_H_ 7.04 (dd, *J* = 8.2, 1.8 Hz, 1H, H-6′), 7.00 (m, 1H, H-5′), and 7.00 (m, 1H, H-2′), and a singlet at δ_H_ 7.42 (s, 1H, H-6). The aliphatic region comprising 4 protons, and they were assigned as H-3 at δ_H_ 5.16 (d, *J* = 8.2 Hz, 1H), H-2 at δ_H_ 4.20 (ddd, *J* = 8.2, 3.7, 2.7 Hz, 1H), H-1″a at δ_H_ 4.06 (dd, *J* = 12.7, 2.7 Hz, 1H), and H-1″b at δ_H_ 3.71 (dd, *J* = 12.7, 3.7 Hz, 1H). Two methoxy singlets were observed at δ_H_ 4.00 (s, OCH_3_, 3H) and 3.96 (s, OCH_3_, 3H), while singlets were observed at δ_H_ 9.79 (s, PhOH, 1H) and δ_H_ 3.51 (s, CH_2_OH, 1H). The ^13^C NMR (Plate S20) and ^13^C APT (Plate S21) spectra displayed 24 carbon signals: nine methines, two methoxy resonances, a carbonyl carbon, eleven quaternary carbons, three methylene carbons, and two methyl carbons as described in Table S9. The 2D COSY and HSQC spectra (Plates S22 and S23) elucidated all proton-proton and proton-carbon correlations. Notable correlations in the 2D HMBC spectrum (Plate S24) include a correlation between H-8 (δ_H_ 8.39) and C-7 (δ_C_ 176.2), C-10 (δ_C_ 134.2), and C-11a (δ_C_ 155.9) (Figure 4). H-9 correlates with C-7a at δ_C_ 121.2, and H-10 correlates with C-8 and C-11a at δ_C_ 126.6 and 155.9, respectively. H-11 correlates with C-9 and C-7a at δ_C_ 123.9 and 121.5, respectively. These correlations confirmed the substitution pattern on the first xanthone ring. H-6 correlates with C-5, C-7, C-4a, and C-12a at δ_C_ 146.8, 176.2, 139.8, and 141.9, respectively, thus confirming the substitution pattern on the second xanthone ring. H-2′ correlates with C-3′ at δ_C_ 147.0 ppm. H-5′ correlates with C-1′ and C-3′ at δ_C_ 126.9 and 147.0, respectively. H-6′ correlates with C-2′ and C-4′ at δ_C_ 109.1 and 146.7, respectively. H-3 correlates with C-2, C-1′, C-2′, and C-6′ at δ_C_ 78.3, 126.9, 109.9, and 121.2, respectively, and H-1″ correlates with C-3. The two methoxy groups correlate with C-5 and C-3′ at δ_C_ 146.8 and 147.0 ppm, respectively.

**Figure 5:**
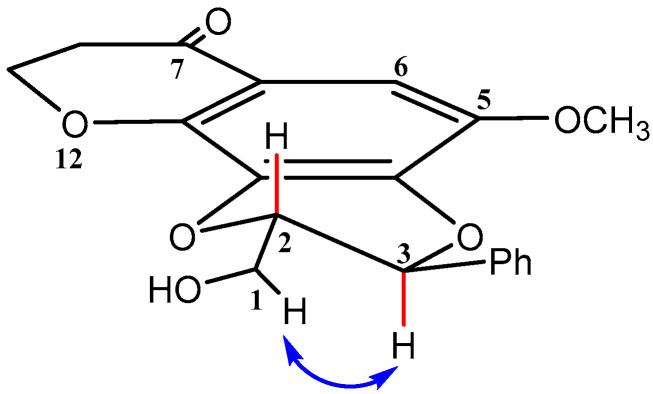
Two-dimensional NOESY correlations of compound **3**.

The relative configuration of the 1,4-dioxane ring was determined via coupling constants and 2D NOESY NMR correlations. The dioxane ring is in a quasi-half-chair conformation. Thus, the relatively large coupling constant between the vicinal H-2 and H-3 (*J* = 8.2 Hz) indicates that the pair is orientated trans-diaxially to each other. The conformational distortion of the dioxane ring leads to a dihedral angle smaller than 180° between H-2 and H-3, explaining the smaller-than-expected *J*-coupling observed (normally 10–14 Hz). Furthermore, the 2D NOESY NMR (Plate S25) displayed a correlation between H-3 and H-1, which will only be possible if they are on the same side of the ring (*cis*) (Figure 5).

Compound **3** was identified as a xanthonolignoid, which comprises a phenylpropane nucleus bonded to a xanthone scaffold by a dioxane moiety and thus was assigned as *trans*-kielcorin, that is 3-(4-hydroxy-3-methoxyphenyl)-2-(hydroxymethyl)-5-metoxy-2,3-dihydro-7*H*-[1,4]dioxino[2,3-*c*]xanthen-7-one. The structure was comfirmed via comparison of the physical data to published data [37,38,39].

In summary, the three compounds isolated from *H. revolutum* include two simple tri-oxygenated xanthones (**1** and **2**), while compound **3** was identified as a kielcorin xanthone (xanthonolignoid derivative). An isomer similar to **1** and **2**, which possesses three methoxy groups with no OH group, was isolated by Abdallah et al. [21], Shiu et al. [40], and Zofou et al. [41]. Cardona et al. [42] obtained compounds **1** and **2** from a different species, *H. reflexum*. Also, Castelão et al. [36] have previously isolated **1** and **2**. As previously stated, this is the first time compounds **1**–**3** were isolated from *H. revolutum*. From NMR integral values, the isolated compounds all have a purity of above 90%.

### 2.3. Bioactivities

#### 2.3.1. α-Glucosidase-Inhibitory Action of Solvent Fractions and Isolated Compounds

**Figure 6:**
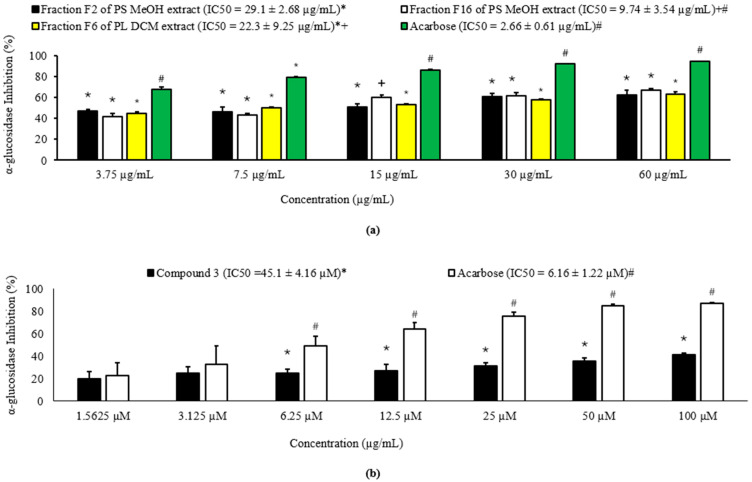
Dose-dependent α-glucosidase-inhibitory action of (**a**) selected fractions of *H. revolutum* and (**b**) compound **3**. Data are shown as mean ± SD of a triplicate experiment. For each concentration, statistical multiple comparisons (Tukey and one-way ANOVA; IBM SPSS) were performed between the treatments. The symbols at the top of the error bars or next to the IC_50_ values of the different treatments mean a significant difference (*p* < 0.05) when the symbols are different between the treatments.

α-Glucosidase is a digestive enzyme in the small intestinal brush border that catalyses the hydrolysis of disaccharides to release glucose [4]. Its inhibition has been shown to suppress glucose absorption and postprandial glycaemia, the mode of action of antidiabetic drugs belonging to the class of drugs known as “α-glucosidase inhibitors” [4]. Three fractions, namely fractions F2 and F16 from the MeOH extract of the PS and fraction F6 from the DCM extract of the PL, showed potent inhibitory action on α-glucosidase activity with 63.5%, 80.8%, and 122.5% inhibitions, respectively (Table 1). The fractions further demonstrated dose-dependent inhibition (IC_50_ = 29.1, 9.74 and 22.3 µg/mL, respectively) of α-glucosidase (Figure 6a), indicating the potency of the fractions to suppress postprandial glycaemia. The fraction F16 from the MeOH extract of the PS was more potent (*p* ˂ 0.05) than the other two fractions and statistically (*p* > 0.05) comparable to acarbose (IC_50_ = 2.66 µg/mL) (Figure 6a).

The data suggests that the above potent fractions from the PS and PL of *H*. *revolutum* may be explored further as possible sources of postprandial glycaemic control compounds. Moreover, some plants belonging to the *Hypericum* genus have been reported to possess α-glucosidase-inhibitory potential [43,44,45].

Compound **3** showed a mild to moderate α-glucosidase-inhibitory activity (IC_50_ = 45.1 ± 4.16 µM) relative to acarbose (IC_50_ = 6.16 ± 1.22 µM) (Figure 6b). The low activity of compound **3** may be attributed to the absence of a hydroxyl group on its xanthone core. A study has shown that the α-glucosidase-inhibitory action of xanthones increases with the number of hydroxyl groups on the xanthone core [46]. Perhaps, chemical modification of compound **3** by transforming the methoxy group at C-5 to a hydroxyl group and cleavage of the ether bond of the xanthone core to yield a free hydroxylated benzene ring may improve its α-glucosidase-inhibitory activity [46]. Although not assessed in our study due, the presence of a hydroxyl group on the xanthone core of compounds **1** and **2** may make them promising targets for future α-glucosidase-inhibitory studies and chemical modifications [46].

#### 2.3.2. Effects on GABA_A_ Receptor Activity

**Figure 7:**
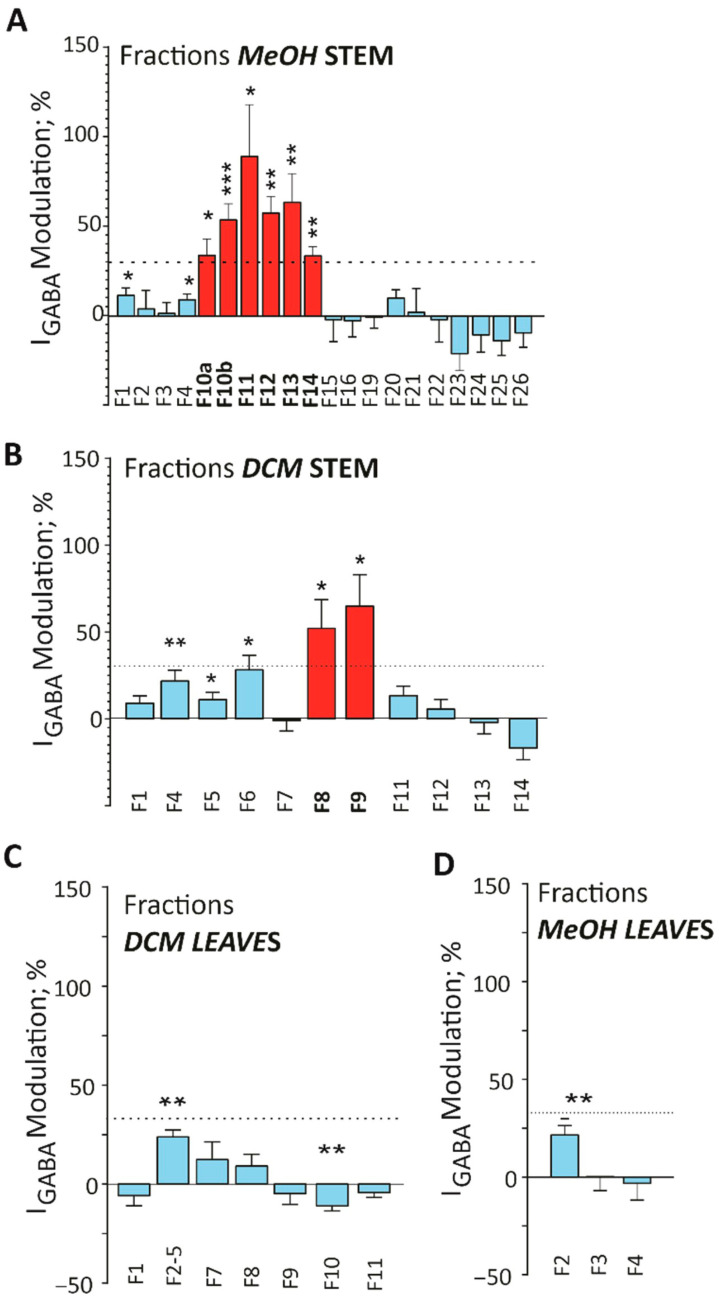
Effects of I_GABA_ by fractions of (**A**) MeOH stem extract and (**B**) DCM stem extract of *H. revolutum*. Subfigures (**C**,**D**) illustrate the I_GABA_ activity of the DCM and MeOH extracts, respectively. Data are shown as mean ± S.E.M from at least 3 oocytes and ≥2 oocyte batches. The dashed line represents the threshold above which a percent change is considered indicative of compound-induced pharmacological effect [47] and statistical significance was calculated using a one-sample *t*-test, with (*) denotes *p* < 0.05, (**) = *p* < 0.01 and (***) = *p* < 0.001.

As shown in Figure 7A,B, several fractions from the PS MeOH and DCM extracts exhibited significant *(p* < 0.05) I_GABA_ enhancement, exceeding the threshold of 30%. In contrast, none of the fractions from the PL MeOH or DCM extracts showed any activity exceeding the 30% threshold (Figure 7C,D).

One-sample *t*-tests confirmed that fractions F8 and F9 of the PS DCM extract significantly increased I_GABA_ by 53 ± 16% (*t* = 3.242, *df* = 6, *p* = 0.0176) and 65 ± 18% (*t* = 3.658, *df* = 6, *p* = 0.0106).

Similarly, one sample *t*-tests identified fractions F10a, F10b, and F11–F14 from the MeOH extract of the PS as active GABA_A_ receptor modulators with activities exceeding 30% I_GABA_ enhancement (Figure 7). Specifically, fraction F10a enhanced I_GABA_ by 34 ± 9% (*t* = 3.873, *df* = 4, *p* = 0.0179), F10b by 54 ± 9%, (*t* = 6.178, *df* = 7, *p* = 0.0005), F11 by 89 ± 29% (*t* = 3.123, *df* = 5, *p* = 0.0262), F13 by 64 ± 16% (*t* = 4.072, *df* = 5, *p* = 0.0096) and F14 by 34 ± 5% (*t* = 7.004, *df* = 4, *p* = 0.0022), respectively. The data suggests that those fractions from the DCM and MeOH extracts of the PS may contain compounds with the potential to modulate GABA_A_ receptors, leading to a calming effect (control of anxiety, stress, excessive fear, and depression).

**Figure 8:**
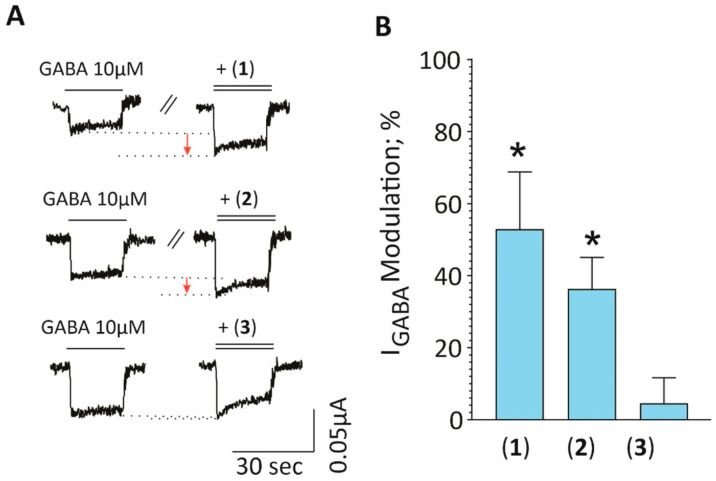
I_GABA_ enhancement by the isolated compounds **1**, **2**, and **3** from *H. revolutum* stems. (**A**) Representative traces illustrating the potentiation of the GABA-induced control current (left current, one bar on top) compared to the modulated current in the presence of the indicated compound (co-application of GABA and compound is indicated by the double bar on top of the trace). (**B**) Bar graphs represent mean ± S.E.M of I_GABA_ modulation derived from 4 to 5 cells from two oocyte batches. Statistical significance was calculated using a one-sample *t*-test, and * denotes *p* < 0.05.

Lastly, we also studied the effects of the isolated xanthone derivatives on I_GABA_ (see Figure 8A for representative current traces). We found that both compounds **1** and **2** significantly potentiated I_GABA_ by 65 ± 13% (t = 4.960, df = 3, *p* = 0.0157) and 36 ± 9% (t = 4.151, df = 3, *p* = 0.0254). In contrast, compound **3** did not significantly affect I_GABA_ (5 ± 7%, *t* = 0.6417, *df* = 4, *p* = 0.5560) (see Figure 8B). It is indeed possible that compound **1** may be responsible for the observed effects of fractions F8 and F9 of the PS DCM extract, since this compound was isolated from both fractions combined. Interestingly, several xanthones, including α-mangostin, have been reported to exhibit antidepressant and anxiolytic effects, which have been linked to their modulatory interaction with GABAergic, serotonergic and dopaminergic systems [48,49,50]. Compound **3**, also a xanthone, did not significantly affect GABA_A_ receptors. This discrepancy may be linked to the observed structural difference when comparing isomeric compounds **1** and **2** to compound **3**. Compound **3** possesses a cyclic ether substitution (Figure 1), which may have impaired a potential interaction with the receptor. Lastly, a one-way ANOVA (*F* (2, 10) = 10.30, *p* < 0.01) followed by a Tukey post hoc mean comparison indicates comparable efficacies of compounds **1** and **2**, suggesting that the position of the hydroxyl group on the xanthone core is not a critical determinant for GABA_A_ receptor activity. It is also important to note that the compounds were tested at 100 µg/mL. While this may not be ideal for compound-to-compound comparison, it does not affect or impact on the findings of our study. This is because the data obtained clearly showed that isomeric compounds **1** and **2** enhanced I_GABA_, while compound **3** did not, suggesting compounds **1** and **2** are potential scaffolds that can be studied further.

This study identified GABA_A_ receptor modulators (compounds **1** and **2**) from *H. revolutum*, demonstrating that *H. revolutum* exhibits central nervous system activity similar to that of *St. John’s wort*. Future studies will investigate their in vivo effects, enabling the assessment of their potential as therapeutic agents for mental disorders such as depression and anxiety.

## 3. Materials and Methods

### 3.1. Materials

The plant stems (PS) and leaves (PL) of *H. revolutum* were identified and harvested by the botanist Dr. Pieter C. Zietsman (ID No: PC and L Zietsman 6654, Bloemfontein Nasional Museum, Bloemfontein, South Africa) at Muilhuis, Blyde River Nature Reserve, Mpumalanga (GPS coordinates: −24.56022, 30.75794) in January 2019. Voucher specimens (Reg No. NMB 27703) are kept at the National Museum in Bloemfontein, South Africa.

The solvents for extraction, fractionation, and isolation were acetone, *n*-hexane, chloroform, dichloromethane, ethyl acetate, and methanol. TLC (thin layer chromatography) was performed on silica gel 60 F254 pre-coated aluminium sheets (Merck, Boston, MA, USA, 0.25 mm—normal phase). Silica gel 600 (Merck, Boston, MA, USA, 0.040–0.063 mm) was used for column chromatography. NMR analysis was performed on a Bruker Avance 600 MHz spectrometer (Bruker, Billerica, MA, USA). High-resolution mass was carried out on an MD Sciex 3200 QTrap equipped with an electrospray (Turbo-ion spray) ionisation source (SCIEX, Framingham, MA, USA). Solid-state Fourier transform infrared spectrometry (FTIR) was performed on a Bruker Tensor 27 spectrometer (Bruker, Billerica, MA, USA).

### 3.2. Solvent Extraction and Qualitative Phytochemical Analyses

Smaller pieces of the PS and PL were air-dried for 21 days at 25 °C. The dried plant parts were ground into a fine powder and yielded: PS 106.04 g and PL 20.26 g. The powdered plant material was consecutively extracted with hexane (Hx) to de-fat the plant material (600 mL, 3 times overnight) [51], followed by dichloromethane (DCM) (600 mL, 3 times overnight), and lastly, methanol (MeOH) (600 mL, 3 times overnight). The extracts were filtered, and the filtrates were concentrated under vacuum. The extracts were air-dried at room temperature to yield the DCM crude extracts (1.6 g for the PS and 0.7 g for the PL) and MeOH crude extracts (8.9 g for the PS and 0.5 g for the PL). The crude extracts were refrigerated [52].

The methanol crude extracts in MeOH (100 mL) were run through a polyamide gel column (CC-6; 50 g, solid-phase extraction, SPE; Phenomenex, Torrance, CA, USA) under gravity flow (MeOH, 2× 250 mL) to remove tannins, indicated by the eluant turning colourless. Concentration under vacuum yielded tannin-free MeOH crude extract (6.1 g PS extract and 0.4 g PL extract) [53].

Qualitative determination of alkaloids [54], saponins [55,56], terpenoids [56], phenols [56], flavonoids [54,57], steroids [55], cardiac glycosides [56], anthraquinones [58], and tannins [57] was performed according to literature procedures.

### 3.3. Fractionation and Isolation of Compounds

Figures S1 and S2 illustrate the isolation process of the active compounds from the stem extract.

The DCM PS crude extract was fractionated via column chromatography using a gradient from Hx:EtOAc, 10:0 until the ratio was slowly adjusted to Hx:EtOAc, 5:5, followed by CHCl_3_:MeOH; 1:1 and then pure methanol. For the MeOH stem crude extract, the gradient was adjusted from Hx: EtOAc 10:0 and gradually adjusted to 0:10, followed by CHCl_3_:MeOH 10:0 to 6:4. The PL extracts were similarly fractionated: the DCM extract from Hx:EtOAc 10:0 to 0:10 and the MeOH extract from CHCl_3_:MeOH, 10:0 to 8:2. Approximately 10 mL of eluent was collected during the fractionation. TLC was used to profile the collected fractions using pre-optimised solvent systems (Tables S1–S5).

Bio-activity-guided isolation was performed on selected fractions using preparative TLC plates (Supplementary Materials) or column chromatography. Compounds **1** and **3** were isolated from the DCM PS extract: fractions F8+F9 (Hx:Ace; 8:2) and F12 (Hx:EtOAc; 5:5), respectively (Figures S1–S3). Sub-fractions of fraction F18 from the MeOH PS extract yielded compound 2 (CHCl_3_:MeOH; 9.5:0.5) (Figures S4 and S5). It is important to note that since very limited isolation studies have been conducted on the plant, all fractions were probed for possible compound isolation, regardless of the bioactivity profiles. Those fractions with good yield and better feasibility of compound isolation were prioritised. Where isolation from a fraction was possible, compounds were isolated and reported.

### 3.4. Spectroscopic and Spezctrometric Characterisation of the Isolated Compounds

High-resolution mass spectroscopy of the isolated compounds was performed using a Waters Synapt G2 qTOF mass spectrometer (Waters Corporation, Milford, MA, USA) equipped with an electrospray (Turbo-ion spray) ionisation source at a 2.5 kV capillary voltage and 15 V cone voltage. The temperature and flow rate of the desolvation gas were 275 °C and 650 L per hour, respectively. The collision energy was 20 V. The scan range was 100–1500 Da. Each sample was manually injected directly into a continuous stream of injection solvent [acetonitrile: 0.1% formic acid (50:50) (*v*/*v*)] at a flow rate of 50 µL min^−1^. Data was acquired and analysed using Masslynx 4.2 software (Waters Corporation, Milford, MA, USA).

The solid-state Fourier transform infrared spectrometry (FTIR) was performed on a Bruker Tensor 27 spectrometer. The FTIR spectra were captured neat between 4000 and 400 cm^−1^.

NMR analysis (600 MHz for ^1^H NMR and 150 MHz for ^13^C NMR) were performed on a Bruker Avance 600 MHz spectrometer having a 5mm DUAL 13C-1H\D probe with z-gradients. The analysis was performed using CDCl_3_ solvent, at a temperature of 25 °C. Tetramethylsilane was used as 0 ppm internal standard. Data analysis was performed using Mnova Software 15.0 (Mestrelab Research, Galicia, Spain).

### 3.5. Function GABA_A_ Receptor Assay

The effects of both fractions and isolated compounds from the PS and PL extracts were determined on GABA-induced chloride currents through the predominantly occurring GABA_A_ receptor subtype (comprising α1, β2, and γ2S subunits). GABA_A_ receptors were heterologously expressed in *Xenopus laevis* (Grade I–II) oocytes as described previously [59,60,61]. The fractions and compounds were tested at a concentration of 100 µg/mL. For the fractions, a 100 µg/mL concentration was used in assessing their I_GABA_ modulatory potential because previous studies [62,63] have also used the same concentration to assess the _IGABA_ modulatory potential of plant extracts and/or fractions in a similar experimental model. For the compounds, we tested their I_GABA_ modulatory potential at 100 µg/mL to maintain a consistent concentration as the fractions, which provides a comparative perspective about the efficacy of the compounds relative to the fractions.

In brief, defolliculated oocytes were obtained from Ecocyte (Dortmund, Germany) and injected with 50–75 nL of nuclease-free water containing a mixture (ratio 1:1:10) of cRNAs encoding for the major GABA_A_ receptor subtype (α1, β2, and γ2S; concentrations ranging between 100 and 2000 ng/μL/subunit) using a Nanoject III microinjector (Drummond Scientific Company, Broomall, PA, USA). Oocytes were stored at 18 °C in ND96 solution composed of 90 mM NaCl, 1 mM KCl, 1 mM MgCl_2_·6H_2_O, 1 mM CaCl_2,_ and 5 mM 2-(4-(2-hydroxyethyl)-1-piperazinyl)ethanesulfonic acid (HEPES), with pH adjusted to 7.4 using 1 M NaOH and supplemented with 100 U/mL penicillin and 10 µg/mL streptomycin. Functional characterisation was then carried out 1–3 days post-injection using two-microelectrode voltage-clamp (TEVC) recordings holding oocytes at −70 mV using a TURBO TEC 03C amplifier (NPI Electronic, Tamm, Germany) connected to an Axon Digidata 1440A digitiser (Molecular Devices, Sunnyvale, CA, USA). Data acquisition was performed using pCLAMP software version 10.3 (Molecular Devices), and current traces were analysed offline. Microelectrodes were filled with 2 M KCl and had resistances between 1 and 3 MΩ. GABA and test compounds were applied using a TECAN Miniprep 60 (npi electronic, Tamm, Germany), permitting semi-automation of the experiments coupled to a fast perfusion system. The oocyte-containing chamber was perfused with 200 µL GABA-containing solutions at a volume rate between 150 and 200 µL/s to elicit chloride currents through GABA_A_ receptors (I*_GABA_*). Modulation of I_GABA_ by the compounds was measured at a GABA concentration eliciting 10% of the maximal current amplitude (EC_10_), peak amplitudes were measured, and the effect was calculated using the following formula:

$$I_{GABA}\;potentiation\left(\%\right)=\left(\frac{Amplitude\;I_{GABA-sample}-Amplitude\;I_{GABA-control}}{Amplitude\;I_{GABA-control}}\right)\times 100$$

I*_GABA+Sample_* is the current induced by co-application of GABA and the tested sample, while I*_GABA−control_* is the current induced by application of GABA alone.

### 3.6. In Vitro α-Glucosidase-Inhibitory Assay

The alpha-glucosidase-inhibitory potency of the fractions was assessed by determining whether the fractions can inhibit the activity of α-glucosidase, a carbohydrate-digesting enzyme. The fractions were tested at a concentration of 60 µg/mL. This concentration was chosen because it falls within the concentration ranges (7.5 to 60 µg/mL and 5.0 to 80 µg/mL) that have been previously used in assessing the digestive enzyme inhibitory potential of plant extracts and/or fractions [64,65]. Three fractions were further subjected to a dose-dependent assay using increasing concentrations (3.75–60 µg/mL) owing to their promising α-glucosidase-inhibitory activity. The fractions included F2 and F16 from the MeOH PS extract and F6 from the DCM PL extract. Compound **3**, isolated from fraction F12 of the DCM PS extract, was subjected to a dose-dependent assay using increasing concentrations (1.5625–100 µM). The activity of the tested samples was compared to the activity of a reference standard (acarbose) at equivalent concentrations. The concentration required to inhibit the enzyme’s activity by 50% (IC_50_) was computed for the dose-dependent tests.

A previous method [66] was adopted to perform the glucosidase inhibition assay. It was performed on a 96-well transparent plate. First, 25 µL of the tested samples or acarbose (at the tested concentrations) or their solvents (control) and 25 µL of a 4 U/mL α-glucosidase solution (dissolved in 100 mM phosphate buffer, pH 6.8) were incubated for 10 min at 37 °C. Next, 50 µL of 10 mM 4-nitrophenyl-β-D-glucopyranoside substrate solution (dissolved in 100 mM phosphate buffer, pH 6.8) was added, and incubation continued for an additional 20 min under the same incubation conditions. After incubation, the enzyme-substrate reaction was stopped by adding 100 µL of a 0.1 M Na_2_CO_3_ solution, and absorbance was measured at 405 nm. The absorbances were blanked using the sample and solvent blanks. The enzyme inhibition activity of the samples was computed using the formula below:

$$Enzyme\;inhibition\;activity\left(\%\right)=\frac{Absorbance\;of\;control-Absorbance\;of\;test}{Absorbance\;of\;control}\times 100$$

### 3.7. Data and Statistical Analysis

For the α-glucosidase-inhibitory assays, data were analysed using the 2016 version of MS Excel and GraphPad Prism 7 (Windows Version). The IC_50_ values were computed as a non-linear fit of transformed (log10) *x*-axis (sample concentration) versus activity (*y*-axis). The data were analysed in triplicate (n = 3) and presented in the average ± standard deviation format. Statistical analysis was performed on the Windows version of IBM SPSS, Version 27 (IBM Corp, Armonk, NY, USA). ANOVA (Tukey post hoc) was used for multiple comparisons of the data averages, while the paired *t*-test was adopted for comparing the data averages of two groups. A statistically significant difference was set at *p* < 0.05 when comparing the mean values of the different groups.

All data from the GABA_A_ receptor assay are expressed as mean ± standard error of the mean (S.E.M) from at least 3 oocytes from 2 to 3 oocyte batches. Statistical analysis was performed using Prism 10 for macOS (Version 10.4.1). One-sample *t*-tests were used to assess drug effects, and *one-way ANOVAs* were employed to detect differences between groups. The threshold for statistical significance was set to *p* < 0.05 for all recordings.

It is important to note that only compound **3** was tested in the enzyme inhibition assay because compounds **1** and **2** were depleted after performing other experiments. The same applies to the fractions that were not tested. Some fractions were completely used up, while trying to isolate compounds, and thus were not tested.

## 4. Conclusions

The glycaemic control potential and effects on the most abundantly expressed GABA_A_ receptor by fractions, as well as the possible bioactive xanthones of *H. revolutum*, were investigated in this study. Xanthones 4-hydroxy-2,3-dimethoxy-9*H*-xanthen-9-one (**1**), 3-hydroxy-2,4-dimethoxy-9*H*-xanthen-9-one (**2**), and *trans*-3-(4-hydroxy-3-methoxyphenyl)-2-(hydroxymethyl)-5-methoxy-2,3-dihydro-7*H*-[1,4]dioxino[2,3-*c*]xanthen-7-one (**3**) were isolated. Although not as potent as acarbose, **3** showed α-glucosidase-inhibitory activity. Compounds **1** and **2** were isomers with potent GABAergic activity. The data suggest that *H. revolutum* stem contains xanthones with α-glucosidase-inhibitory and GABAergic activity, which may be useful for both diabetic patients and depression.

## Figures and Tables

**Table 1:** In vitro α-glucosidase-inhibitory activities of fractions from the MeOH and DCM extracts of *H. revolutum* stem and leaves tested at 60 μg/mL.

Fractions from MeOH Extract of PS	Glucosidase-Inhibitory Activity (%)	Fractions from DCM Extract of PS	Glucosidase-Inhibitory Activity (%)	Fractions from MeOH Extract of PL	Glucosidase-Inhibitory Activity (%)	Fractions from DCM Extract of PL	Glucosidase-Inhibitory Activity (%)
F1	36.6 ± 6.35	F1	51.07 ± 8.08	F1	9.89 ± 0.42	F1	39.7 ± 2.47
F2	63.5 ± 3.74	F4	32.0 ± 4.64	F2	20.5 ± 1.92	F6	122 ± 9.55
F3	NAD	F5	34.3 ± 6.09	F3	19.8 ± 2.00	F7	NAD
F4	10.8 ± 2.64	F6	17.4 ± 3.07	F4	22.2 ± 9.76	F8	6.76 ± 1.46
F10a	NAD	F7	17.3 ± 5.38			F9	NAD
F11	NAD	F9	20.1 ± 5.40			F10	22.1 ± 3.65
F12	NAD	F11	2.44 ± 0.18			F11	8.90 ± 3.44
F13	NAD	F12	23.4 ± 6.97				
F14	NAD	F13	NAD				
F15	8.63 ± 0.18	F14	21.5 ± 4.41				
F16	80.8 ± 14.1						
F18	NAD						
F19	NAD						
F20	22.6 ± 10.5						
F21	NAD						
F22	25.3 ± 6.77						
F23	21.3 ± 0.18						
F24	21.5 ± 5.43						
F25	12.2 ± 4.05						
F26	9.99 ± 3.85						
Acarbose	94.3 ± 1.86						

DCM: Dichloromethane; MeOH: Methanol; NAD: No activity detected; PL: Plant leaves; PS: Plant stem.

## Data Availability

Data will be made available on request.

## Supplementary Materials

The following supporting information can be downloaded at: https://www.mdpi.com/article/10.3390/molecules30173530/s1, Figure S1: TCL plates showing the fractions and isolated compound **1**; Figure S2: TCL plates showing the fractions and isolated compound **3**; Figure S3: Schematic representation of the isolation of compounds **1** and **3** from the DCM extract of *H. revolutum* stems; Figure S4: TCL plates showing the fractions and isolated compound **2**. Figure S5: Schematic representation of the isolation of compound **2** from the MeOH extract of *H. revolutum* stems; Table S1: Phytochemical analysis of the DCM and MeOH crude extracts of PS; Table S2: Gradient fractionation of the DCM extract of the plant stems; Table S3: Gradient fractionation of the MeOH extract of the plant stems; Table S4: Sub-Fractions from fraction 18 of the MeOH extract of the plant stems; Table S5: Gradient fractionation of the DCM extract of the plant leaves; Table S6: Gradient fractionation of the MeOH extract of the plant leaves; Table S7: ^1^H and ^13^C NMR data of compound **1** [600 MHz, CDCl_3_, δ (ppm), J (Hz)]; Table S8: ^1^H and ^13^C NMR data of compound **2** [600 MHz, CDCl_3_, δ (ppm), J (Hz)]; Table S9: ^1^H and ^13^C NMR data of compound **3** [600 MHz, CDCl_3_, δ (ppm), J (Hz)]; Plate S1: Plate S1 HR-ESI MS spectrum of Compound **1**; Plate S2: Infrared spectrum of Compound **1**; Plate S3: ^1^H NMR (600 MHz) spectrum of Compound **1** CDCl_3_; Plate S4: ^13^C NMR (150 MHz) spectrum of Compound **1** (CDCl_3_); Plate S5: ^13^C APT NMR (150 MHz) spectrum of Compound **1** (CDCl_3_); Plate S6: 2D COSY NMR spectrum of Compound **1** (CDCl_3_); Plate S7: 2D HSQC spectrum of Compound **1** (CDCl_3_); Plate S8: 2D HMBC spectrum of Compound **1** (CDCl_3_); Plate S9: HR-ESI MS spectrum of Compound **2**; Plate S10: Infrared spectrum of Compound **2**; Plate S11: ^1^H NMR (600 MHz) spectrum of Compound **2** CDCl_3_; Plate S12: ^13^C NMR (150 MHz) spectrum of Compound **2** (CDCl_3_); Plate S13: ^13^C APT NMR (150 MHz) spectrum of Compound **2** (CDCl_3_); Plate S14: 2D COSY NMR spectrum of Compound **2** (CDCl_3_); Plate S15: 2D HSQC spectrum of Compound **2** (CDCl_3_); Plate S16: 2D HMBC spectrum of Compound **2** (CDCl_3_); Plate S17: Plate S1 HR-ESI MS spectrum of Compound **3**; Plate S18: Infrared spectrum of Compound **3**; Plate S19: ^1^H NMR (600 MHz) spectrum of Compound **3** CDCl_3_; Plate S20: ^13^C NMR (150 MHz) spectrum of Compound **3** (CDCl_3_); Plate S21: ^13^C APT NMR (150 MHz) spectrum of Compound **3** (CDCl_3_); Plate S22: 2D COSY NMR spectrum of Compound **3** (CDCl_3_); Plate S23: 2D HSQC spectrum of Compound **3** (CDCl_3_); Plate S24: 2D HMBC spectrum of Compound **3** (CDCl_3_); Plate S25: 2D NOESY spectrum of Compound **3** (CDCl_3_).

## Abbreviations

DCM	dichloromethane
EtOAc	ethyl acetate
FTIR	Fourier transform infrared spectrometry
GABA	gamma-aminobutyric acid
Hx	hexane
I_GABA_	GABA-induced chloride currents
MeOH	methanol
NMR	nuclear magnetic resonance
PL	plant leaves
PS	plant stem
TLC	thin layer chromatography
