# Supplementary material for: GABAergic and α-Glucosidase-Inhibitory Potentials of Fractions and Isolated Xanthones from Hypericum revolutum Vahl subsp. revolutum
Source: Molecules. 2025 Aug 29;30(17):3530. doi: 10.3390/molecules30173530 (PMC12430133; doi:10.3390/molecules30173530)

SUPPLEMENTARY MATERIAL

The supplementary materials are grouped into Tables (Tables S1 – S8), Figures (Figures S1 – S5) and Plates (Plates S1 – S25)

TABLES (TABLE S1 – S9)

Preparative TLC plates and Thin Layer Chromatography Analysis

After the fractions were plotted again on the aluminium TLC plates and silica gel 60 F254 pre-coated aluminium sheets (Merck, 0.25 mm – normal phase) for proper identification. F8 and F9 showed the spots on the TLC plates, hence, were combined together and named F8. F8 was dissolved in acetone and uniformly poured on the preparative TLC plate in a single line form on the bottom part of the preparative TLC plate, allowed to dry, then placed in a TLC chamber containing n-hexane:Ace; 8:2 solvent mixture, was allowed to elute. After 15 minutes of full absorption to the top of the plate, the plates were removed, allowed to dry and **A** was spotted under UV light and was scrapped out from the plates, then thoroughly washed with acetone, dried and weighed. Hence, compound **A** was obtained. The same was applicable on the other two isolated compounds using their respective solvent ration as stated. The isolated compounds were spotted on the TLC plates (just a cm from the bottom) again for a perfect view, was allowed to dry and was placed in a chamber that contains the same solvent ratio used for the isolation, was allowed to elute. TLC showed a perfect separation of the isolated compounds under UV light at wavelengths of 254 and 366 nm as blue spots for each compound, afterwards the Rf values were calculated.

Table S1. Phytochemical analysis of the DCM and MeOH crude extracts of PS.

Phytoconstituents	Test	Conclusion	
		DCM	MeOH
Alkaloids	Dragendorff's reagent	+++	+++
Anthraquinones	**Borntrager**	---	++
Cardiac glycosides	Kellar-Kiliani	+++	+++
Flavanoids	Alkaline	---	+++
	Lead acetate	---	+++
Saponins	Froth test	---	+++
	Foam test	+++	---
Steroids	Liebermann-Burchardt	---	++
Phenols	Ferric chloride	+++	+++
Terpenoids	Salkowski	----	++
Tannins	Ferric chloride	---	---

(++) Moderate presence), (+++) Abundant present, (---) Absent

Fractionation, solvent systems, and yield.

Table S2: Gradient fractionation of the DCM extract of the plant stems.

Fr. number	Eluent system	Ratio	Mass (g)
1	Hx	10	0.0351
	Hx:EtOAc	9:1	
2	Hx:EtOAc	8:2	0.0048
3	Hx:EtOAc	8:2	0.0139
4	Hx:EtOAc	8:2	0.0256
5	Hx:EtOAc	7:3	0.0435
6	Hx:EtOAc	7:3	0.0204

Fr. number	Eluent system	Ratio	Mass (g)
7	Hx:EtOAc	7:3	0.0291
8	Hx:EtOAc	7:3	0.0136
9	Hx:EtOAc	7:3	0.0256
10	Hx:EtOAc	6:4	0.0906
11	Hx:EtOAc	5:5	0.0490
12	$CHCl_3$:MeOH	1:1	0.4901
13	$CHCl_3$:MeOH	1:1	0.1283
14	$CHCl_3$:MeOH	0:10	0.0206

Table S3: Gradient fractionation of the MeOH extract of the plant stems.

Fr. number	Eluent system	Ratio	Mass (g)	Fr. number	Eluent system	Ratio	Mass (g)
1	Hx	10:0	0.0167				
	Hx:EtOAc	9:1		14	Hx:EtOAc	1:9	0.0227
2	Hx:EtOAc	8:2	0.0076	15	Hx:EtOAc	0:10	0.0202
3	Hx:EtOAc	8:2	0.0070	16	$CHCl_3$:MeOH	10:0	0.0605
4	Hx:EtOAc	8:2	0.0049	17	$CHCl_3$:MeOH	10:0	0.0163
5	Hx:EtOAc	7:3	0.0020	18	$CHCl_3$:MeOH	9.5:0.5	0.1234
6	Hx:EtOAc	7:3	0.0031	19	$CHCl_3$:MeOH	9:1	0.0564
7	Hx:EtOAc	7:3	0.0034	20	$CHCl_3$:MeOH	9:1	0.0331
8	Hx:EtOAc	7:3	0.0047	21	$CHCl_3$:MeOH	8:2	0.1138
9	Hx:EtOAc	7:3	0.0083	22	$CHCl_3$:MeOH	8:2	0.3826
10	Hx:EtOAc	6:4/5:5	0.0255	23	$CHCl_3$:MeOH	7.5:2.5	0.0377
11	Hx:EtOAc	4:6	0.0134	24	$CHCl_3$:MeOH	7.5:2.5	0.2821
12	Hx:EtOAc	3:7	0.0094	25	$CHCl_3$:MeOH	7:3	0.3200
13	Hx:EtOAc	2:8	0.0133	26	$CHCl_3$:MeOH	6:4	0.2978

Table S4: Sub-Fractions from fraction 18 of the MeOH extract of the plant stems.

Fr. number	Eluent system	Ratio	Mass (g)
18.1	$CHCl_3$:MeOH	9.5:0.5	0.0171
18.2	$CHCl_3$:MeOH	9.5:0.5	0.0240
18.3	$CHCl_3$:MeOH	9.5:0.5	0.0020
18.4	$CHCl_3$:MeOH	9.5:0.5	0.0240
18.5	$CHCl_3$:MeOH	9.5:0.5	0.0074
18.6	$CHCl_3$:MeOH	9.5:0.5	0.0360
18.7	$CHCl_3$:MeOH	9.5:0.5	0.0072
18.8	$CHCl_3$:MeOH	9.5:0.5	0.0790
18.9	$CHCl_3$:MeOH	9.5:0.5	0.0015
18.10	$CHCl_3$:MeOH	0:10	0.0044

Table S5: Gradient fractionation of the DCM extract of the plant leaves.

Fr. number	Eluent system	Ratio	Mass (g)
1	Hexane	10	0.0106
2	Hx:EtOAc	8:2	0.0110
3	Hx:EtOAc	7:3	0.0035
4	Hx:EtOAc	7:3	0.0197
5	Hx:EtOAc	6:4	0.0201
6	Hx:EtOAc	5:5	0.0015
7	Hx:EtOAc	4:6	0.0060
8	Hx:EtOAc	3:7	0.0451

Fr. number	Eluent system	Ratio	Mass (g)
9	Hx:EtOAc	2:8	0.0160
10	Hx:EtOAc	1:9	0.0123
11	Hx:EtOAc	0:10	0.0406

Table S6: Gradient fractionation of the MeOH extract of the plant leaves.

Fr. number	Eluent system	Ratio	Mass (g)
1	$CHCl_3$:MeOH	10	0.0035
2	$CHCl_3$:MeOH	9:1	0.0087
3	$CHCl_3$:MeOH	8:2	0.0084
4	$CHCl_3$:MeOH	8:2	0.0066

Table S7: ^{1}H and ^{13}C NMR data of compound **1** [600 MHz, $CDCl_3$, δ (ppm), J (Hz)].

Position	δ_H Experimental (ppm) (Mult.)	δ_C Experimental (ppm)	δ_C (Published) (ppm) (Castelão *et al.*, 1977)
1	7.36 (s, 1H)	97.7 CH	95.5 CH
2	-	149.4 C	149.8 C
3	-	141.1 C	142.1 C
4	-	138.0 C	139.5 C
4a	-	137.9 C	141.6 C
5	7.60 (dd, *J* = 8.5, 1.1 Hz, 1H)	117.9 CH	118.0 CH
6	7.72 (ddd, *J* = 8.5, 7.1, 1.7 Hz, 1H),	134.3 CH	134.6 CH
7	7.39 (ddd, *J* = 8.1, 7.1, 1.1 Hz, 1H)	123.9 CH	123.8 CH
8	8.35 (dd, *J* = 8.1, 1.7 Hz, 1H)	126.6 CH	125.7 CH
8a	-	121.3 C	120.4 C
9	-	176.4 C=O	174.5 C=O
9a	-	117.6 C	116.7 C
10a	-	155.9 C	155.3 C

OMe	4.08 (s, 3H)	56.1 C-OMe	55.7 C-OMe
OMe	3.98 (s, 3H)	61.4 C-OMe	60.5 C-OMe
OH	6.06 (s, 1H)	-	-

Table S8: ^{1}H and ^{13}C NMR data of compound **2** [600 MHz, $CDCl_3$, δ (ppm), J (Hz)].

**Positions**	**δ ^{1}H Experimental (ppm) (Mult.)**	**δ_C Experimental (ppm)**	**δ_C (Published) (ppm)** (João *et al.,* 1977)
1	7.53 (s, 1H)	100.6 CH	99.8 CH
2	-	145.0 QC	146.7 C
3	-	144.8 QC	149.1 C
4	-	134.8 QC	135.0 C
4a	-	146.1 QC	146.1 C
5	7.57 (dd, *J* = 8.5, 1.1 Hz, 1H)	117.9 CH	117.8 CH
6	7.73 (ddd, *J* = 8.5, 7.1, 1.7 Hz, 1H)	134.1 CH	134.0 CH
7	7.39 (ddd, *J* = 8.1, 7.1, 1.1 Hz)	123.9 CH	123.7 CH
8	8.35 (dd, *J* = 8.1, 1.7 Hz, 1H)	126.6 CH	125.6 CH
8a	-	121.3 QC	120.7 C
9	-	176.1 C=O	174.1 C=O
9a	-	114.6 QC	111.6 C
10a	-	155.9 QC	155.1 C
OMe	4.13 (s, 3H)	56.5 C-OMe	55.8 C-OMe
OMe	4.03 (s, 3H)	61.7 C-OMe	60.8 C-OMe
OH	6.27 (brd s, 1H)	-	-

Table S9: ^{1}H and ^{13}C NMR data of compound **3** [600 MHz, $CDCl_3$, δ (ppm), J (Hz)].

**Proton**	**δH** Experimental (ppm) (Mult.)	**δC** Experimental (ppm)	**δH** (Publ)* (ppm)	**δC** (Publ)* (ppm)	**δH** (Publ)# (ppm)	**δC** (Publ)# (ppm)	**δH** (Publ)† (ppm)	**δC** (Publ)† (ppm)
1″a	3.71 (dd, *J* = 12.7, 3.7 Hz)	61.4 CH_2	3.75 (dd, *J* = 12, 3.6 Hz, 1H)	59.8 CH_2	3.5 (m)	60.7 CH_2	3.47 (m)	59.8 CH_2
1″b	4.06 (dd, *J* = 12.7, 2.7 Hz)	61.4 CH_2	3.75 (dd, *J* = 12, 3.6 Hz)	59.8 CH_2	3.9 (m)	60.7 CH_2	3.75 (m)	59.8 CH_2
2	4.20 (ddd, *J* = 8.2, 3.7, 2.7 Hz, 1H)	78.3 CH	4.39 (m, *J* = 8 Hz, 1H)	77.8 CH	4.1 (m)	77.3 CH	4.43-4.40 (m)	77.8 CH
3	5.16 (d, *J* = 8.2 Hz, 1H)	77.1 CH	5.07 (d, *J* = 8 Hz, 1H)	76.3 CH	5.1 (d, *J* = 8.1 Hz, 1H)	78.4 CH	5.10 (d, *J* = 8.0 Hz, 1H)	76.4 CH
4a	-	139.8 QC	-	139.4 QC	-	139.2		139.6 QC
5	-	146.2 QC	-	145.7 QC	-	146.4		145.8 QC
6	7.42 (s, 1H)	97.6 CH	7.17 (s, 1H)	96.4 CH	7.33 (s, 1H)	98.3 CH	7.23 (s, 1H)	96.5 CH
6a	-	115.0 QC	-	115.4 QC	-	118.0		113.9 QC
7	-	176.2 C=O	-	174.6 C=O	-	176.7 C=O		174.7 C=O
7a	-	121.5 QC	-	120.8 QC	-	123.8 QC		120.7 QC
8	8.39 (dd, *J* = 8.0, 1.7 Hz, 1H)	126.6 CH	8.18 (dd, *J* = 7.2 Hz, 1H)	125.7 CH	8.2 (dd, *J* = 7.3. 1.4 Hz, 1H)	126.5 CH	8.22 (dd, *J* = 7.5, 1.5)	125.8 CH
9	7.43 (td, *J* = 8.0, 7.0, 1.0 Hz, 1H)	123.9 CH	7.47 (td, *J* = 7.2 Hz, 1H)	124.1 CH	7.42 (t, *J* = 7.3 Hz, 1H)	125.3 CH	7.49 (dd, *J* = 7.5, 7.5 Hz, 1H)	124.2 CH
10	7.74 (td, *J* = 8.5, 7.0, 1.7 Hz, 1H)	134.2 CH	7.84 (td, *J* = 7.2 Hz, 1H)	134.5 QC	-	134.5 CH	7.86 (dd, *J* = 7.5, 7.5 Hz, 1H)	134.8 CH
11	7.61 (dd, *J* = 8.5, 1.0 Hz, 1H)	118.0 CH	7.66 (dd, 9.2 Hz, 1H)	117.9 CH	7.62 (d, *J* = 8.4 Hz, 1H)	119.8 CH	7.70 (d, *J* = 7.5, 1H)	118.0 CH

**Proton**	**δ$_H$** Experimental (ppm) (Mult.)	**δ$_C$** Experimental (ppm)	**δ$_H$** (Publ)* (ppm)	**δ$_C$** (Publ)* (ppm)	**δ$_H$** (Publ)# (ppm)	**δ$_C$** (Publ)# (ppm)	**δ$_H$** (Publ)† (ppm)	**δ$_C$** **(**Publ)† (ppm)
11a	-	155.9 QC	-	155.2 QC	-	155.4 QC		155.3 QC
12a	-	141.9 QC	-	142.5 QC	-	142.0 QC		141.2 QC
12b	-	132.4 QC	-	132.4 QC	-	-		132. QC5
1′	-	126.9 QC	-	126.6 QC	-	-		126.6 QC
2′	(m, 1H)	109.9 CH	7.09 (br s, 1H)	112.1 CH	7.04 (d, *J* = 1.4 Hz, 1H)	112.9 CH	7.08 (d, *J* = 1.5, 1H)	112.1 CH
3′	-	147.0 QC	-	147.6 QC	-	147.0		147.6 QC
4′	-	146.7 QC	-	147.3 QC	-	-		147.3 QC
5′	6.85-7.00 (m, 1H)	114.9 CH	6.86 (d, *J* = 8, Hz, 1H)	113.8 CH	6.8 (d, *J* = 6.2 Hz, 1H)	116.8 CH	6.85 (d, *J* = 8.0, 1H)	115.4 CH
6′	7.04 (dd, *J* = 8.2, 1.8 Hz, 1H)	121.2 CH	6.93 (dd, *J* = 8, Hz, 1H),	120.6 CH	6.9 (dd, *J* = 6.1, 1.4 Hz, 1H)	122.9 CH	6.92 (dd, *J* = 8.0, 1.5, 1H)	120.8 CH
OMe	4.00 (s, 3H)	56.3 C-OCH_3	3.86 (s, 3H)	56.7 C-OCH_3	3.84 (s, 3H)	56.1 C-OCH_3	3.88 (s, 3H)	55.7 C-OCH_3
OMe	3.96 (s, 3H)	56.2 C-OCH_3	3.81 (s, 3H)	56.6 C-OCH_3	3.89 (s, 3H)	56.9 C-OCH_3	3.60 (s, 3H)	55.8 C-OCH_3
CH_2**OH**	3.51 (s, 1H)	-	5.16 (s, CH_2OH)	-	-	-		
Ph**OH**	9.79 (s, 1H)	-	9.29 (s, PhOH)	-	-	-	9.22 (s, PhOH)	

*Mesquita *et al*., 1987; #Ali et al., 2011; †Coqueiro et al., 2016

FIGURES (FIGURES S1 – S5)

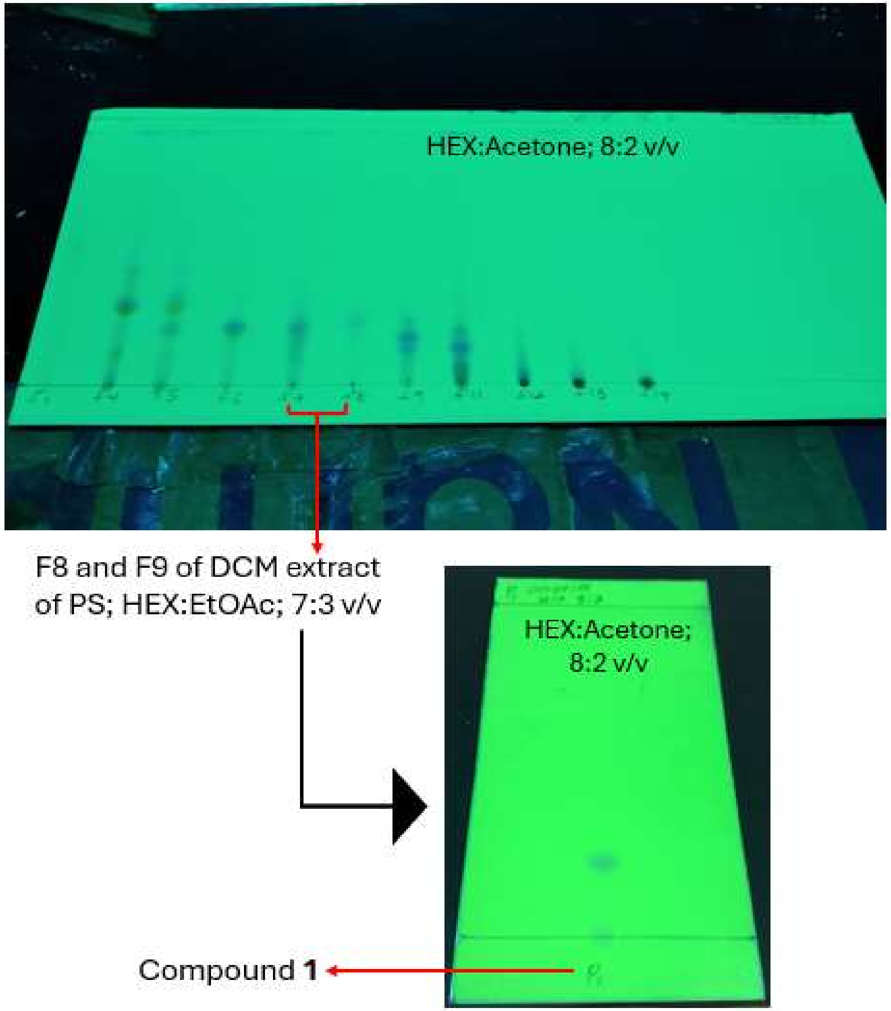

Figure S1: TCL plates showing the fractions and isolated compound 1.

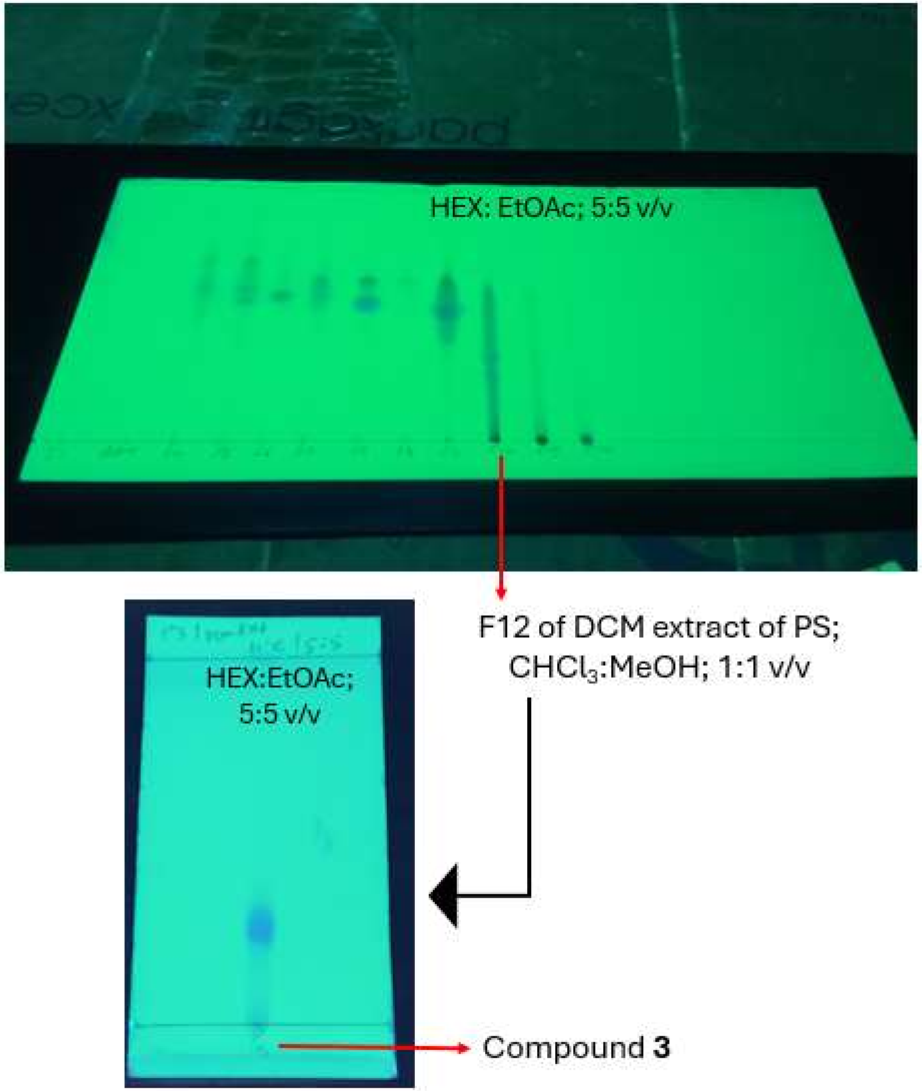

Figure S2: TCL plates showing the fractions and isolated compound 3.

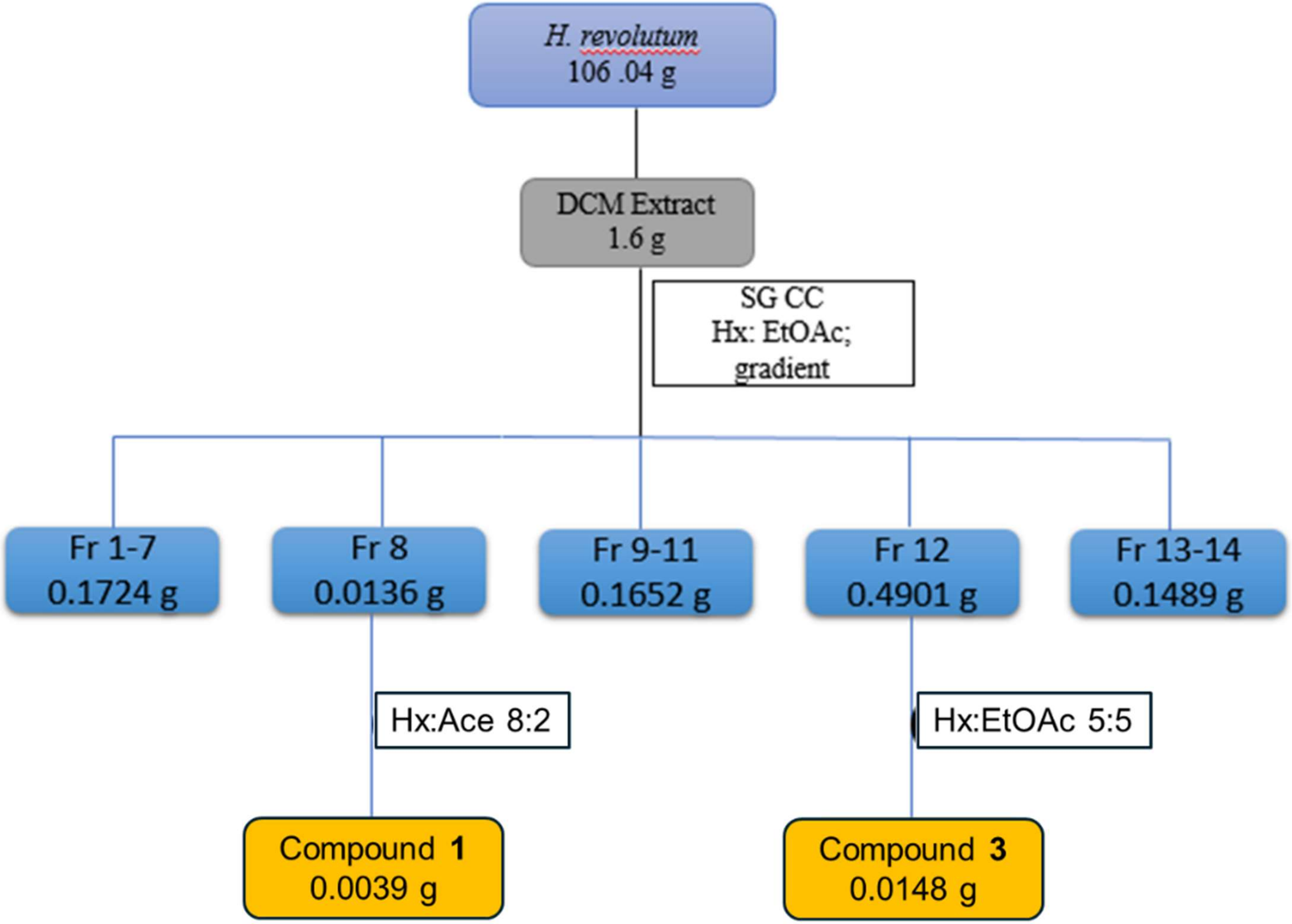

Figure S3: Schematic representation of the isolation of compounds 1 and 3 from the DCM extract of *H. revolutum* stems.

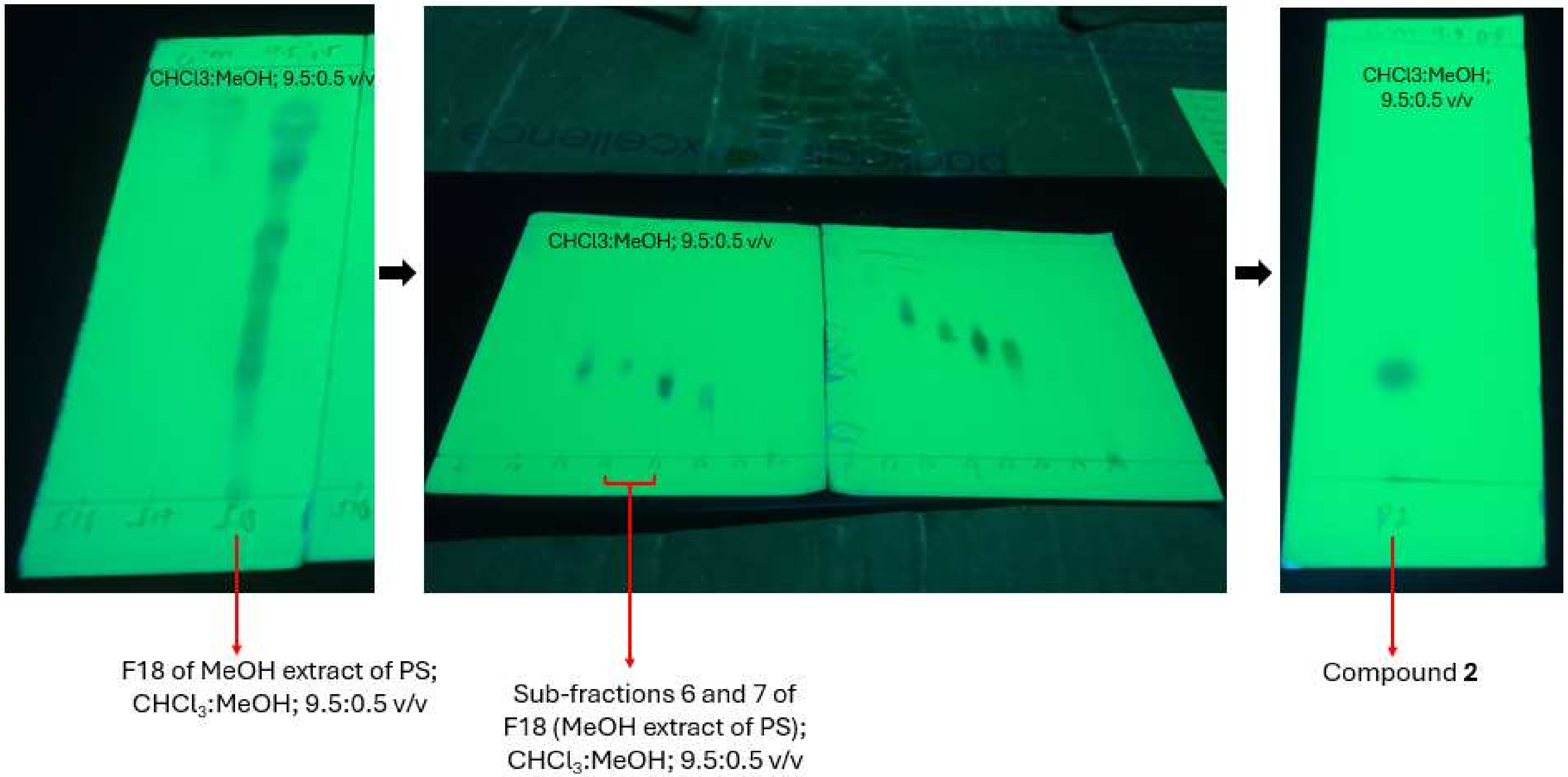

F18 of MeOH extract of PS;
$CHCl_3$:MeOH; 9.5:0.5 v/v

Sub-fractions 6 and 7 of
F18 (MeOH extract of PS);
$CHCl_3$:MeOH; 9.5:0.5 v/v

Compound **2**

Figure S4: TCL plates showing the fractions and isolated compound 2.

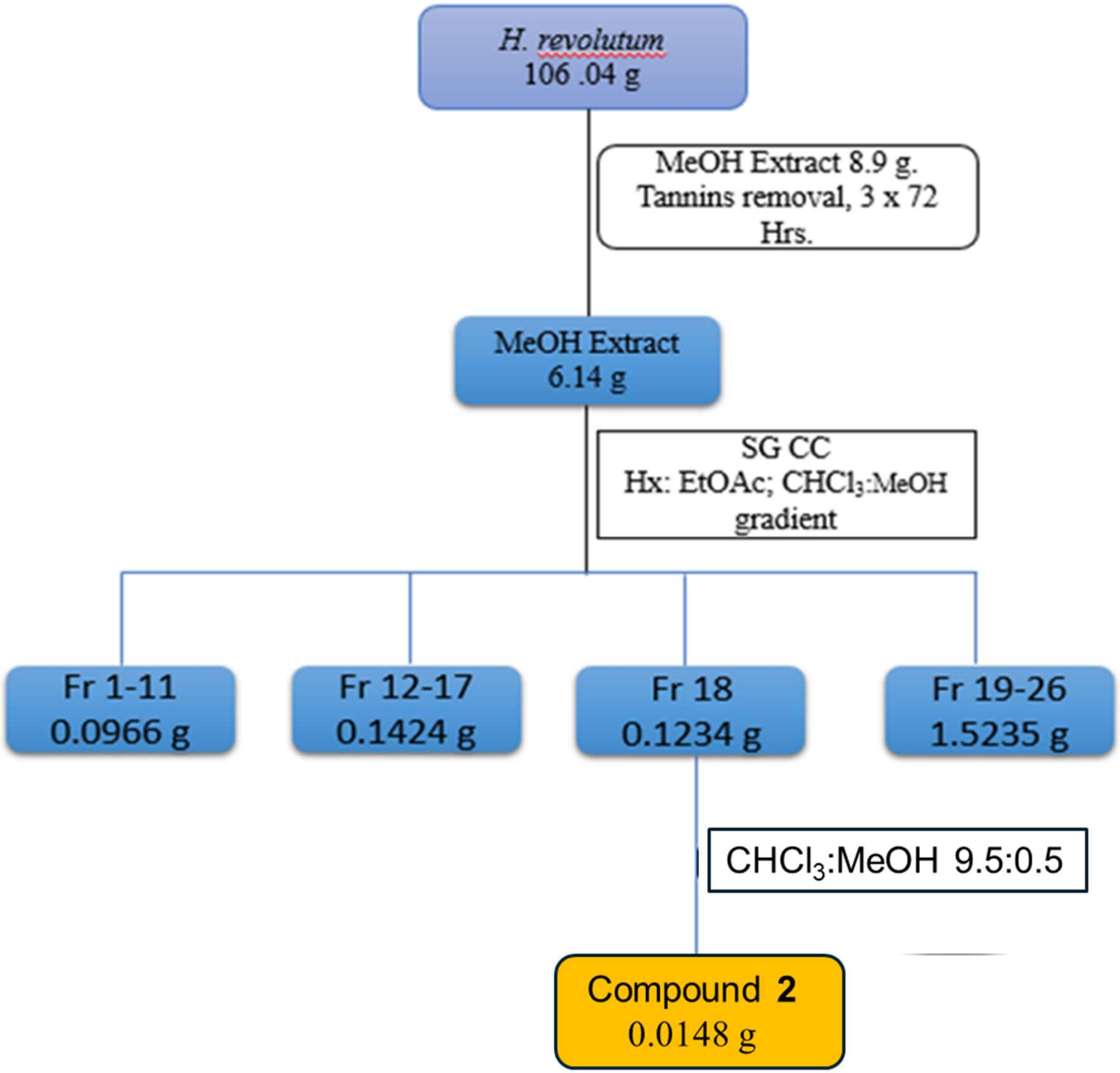

Figure S5: Schematic representation of the isolation of compound 2 from the MeOH extract of *H. revolutum* stems.

PLATES (PLATES S1 – S25)

Plate S1 HR-ESI MS spectrum of Compound 1

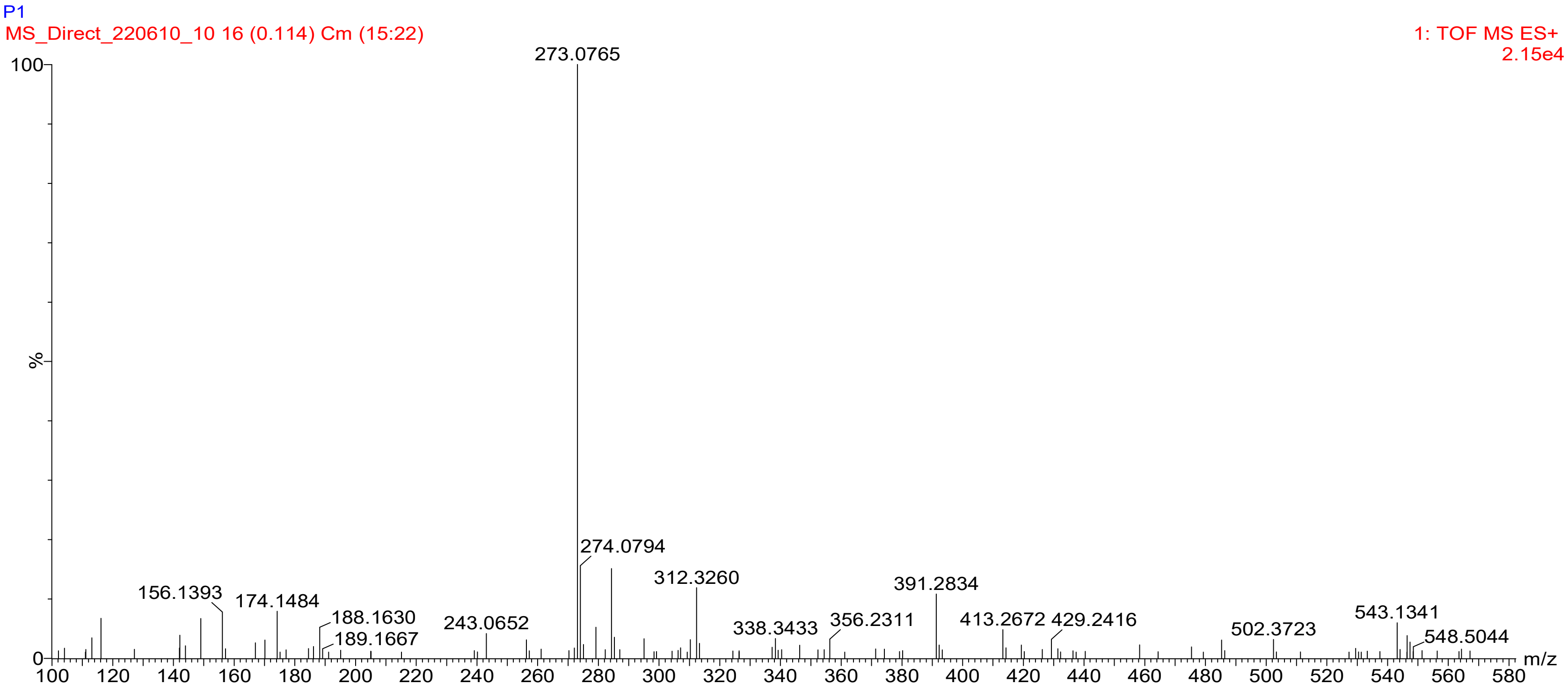

Plate S2 Infrared spectrum of Compound 1

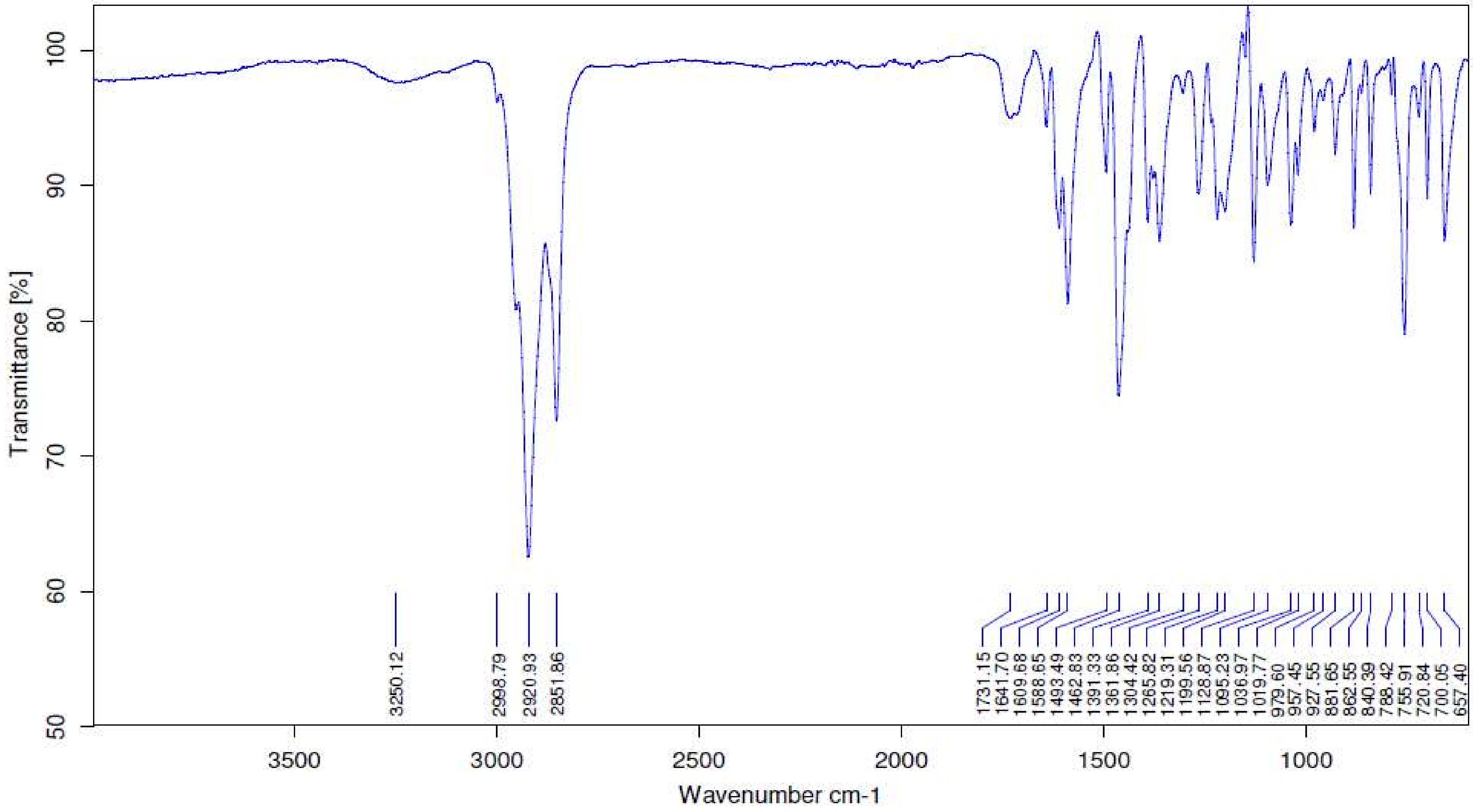

Plate S3 ^{1}H NMR (600 MHz) spectrum of Compound **1** $CDCl_3$

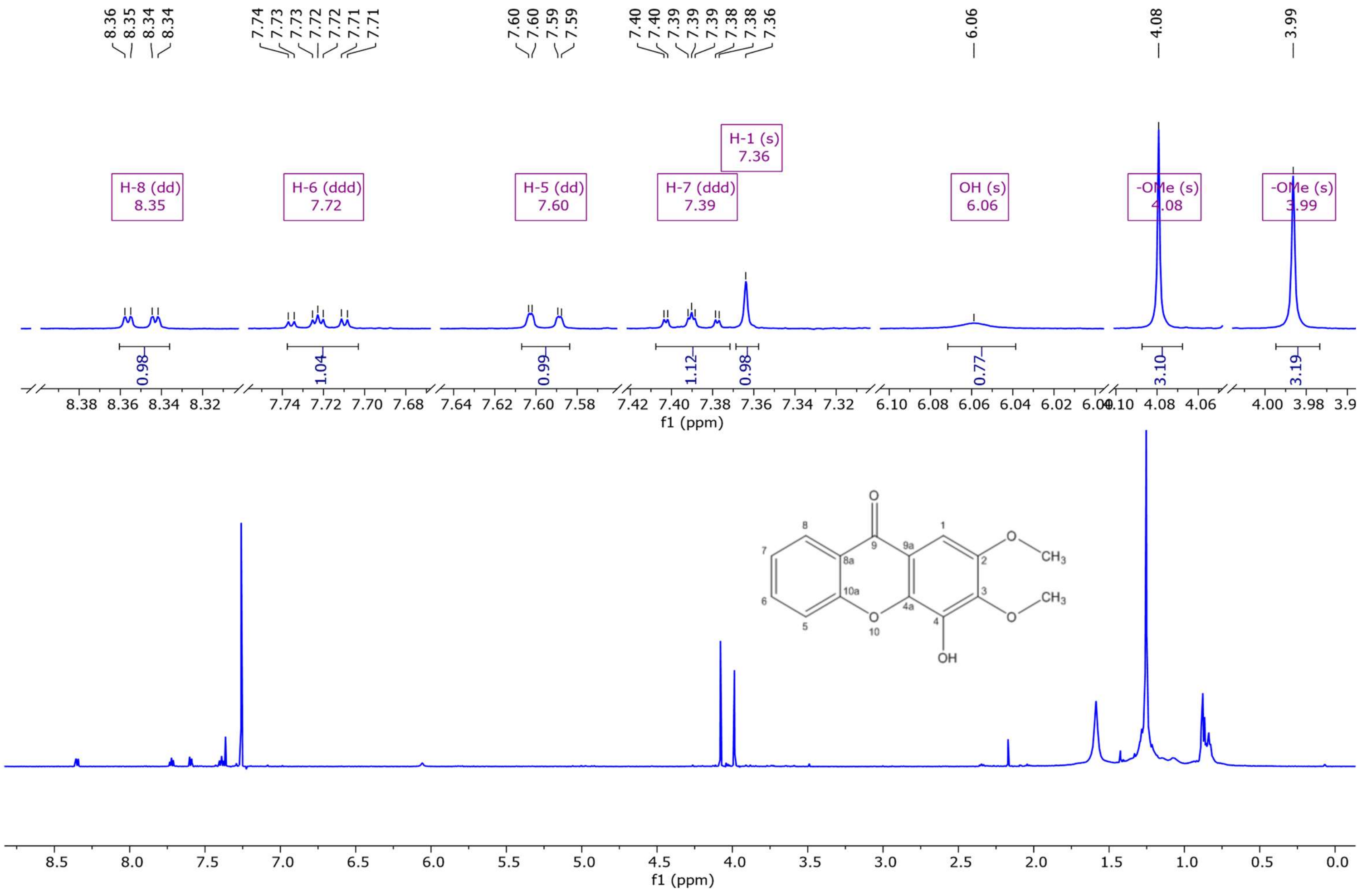

Plate S4 ^{13}C NMR (150 MHz) spectrum of Compound **1** ($CDCl_3$)

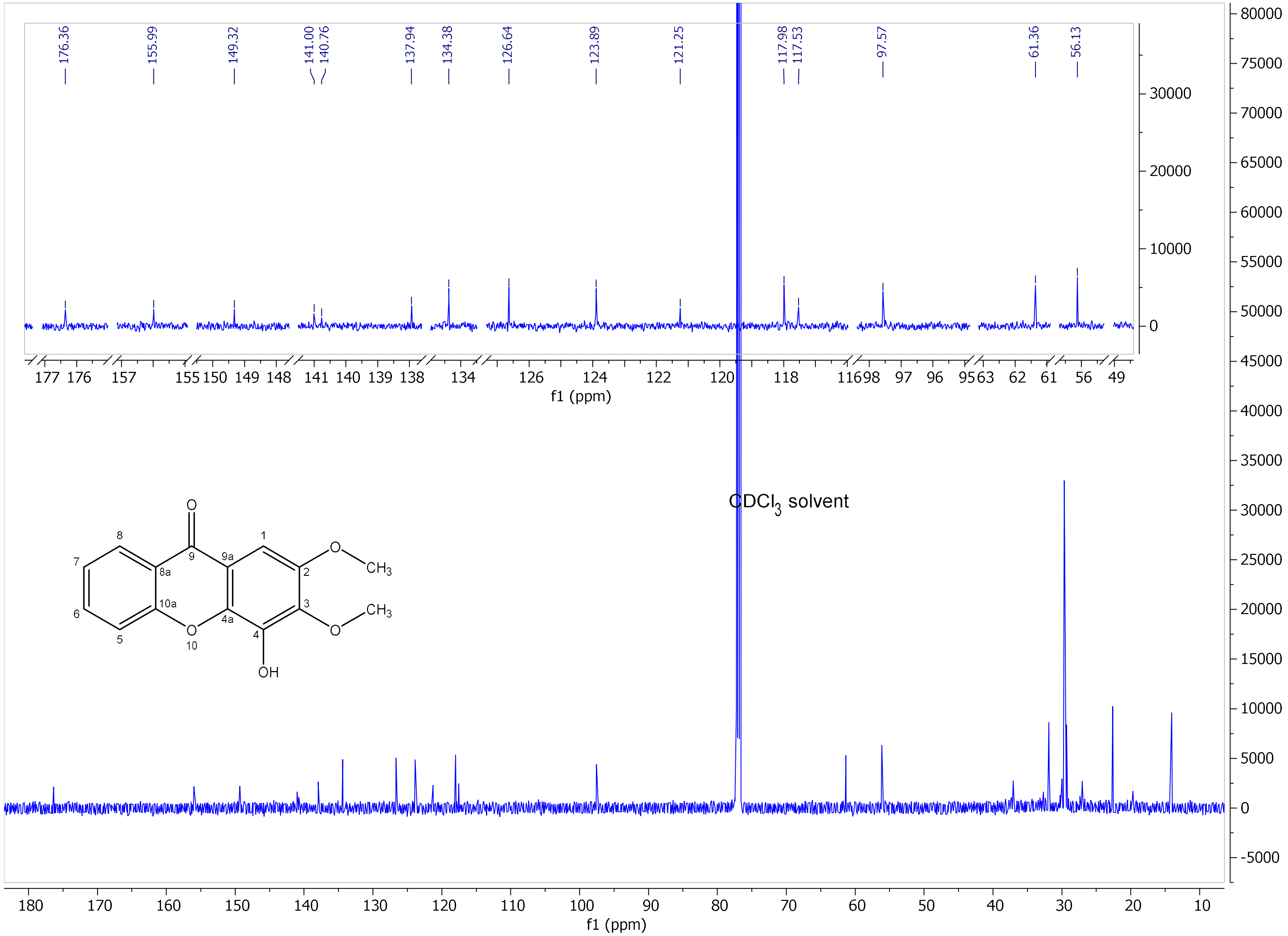

Plate S5 ^{13}C APT NMR (150 MHz) spectrum of Compound **1** ($CDCl_3$)

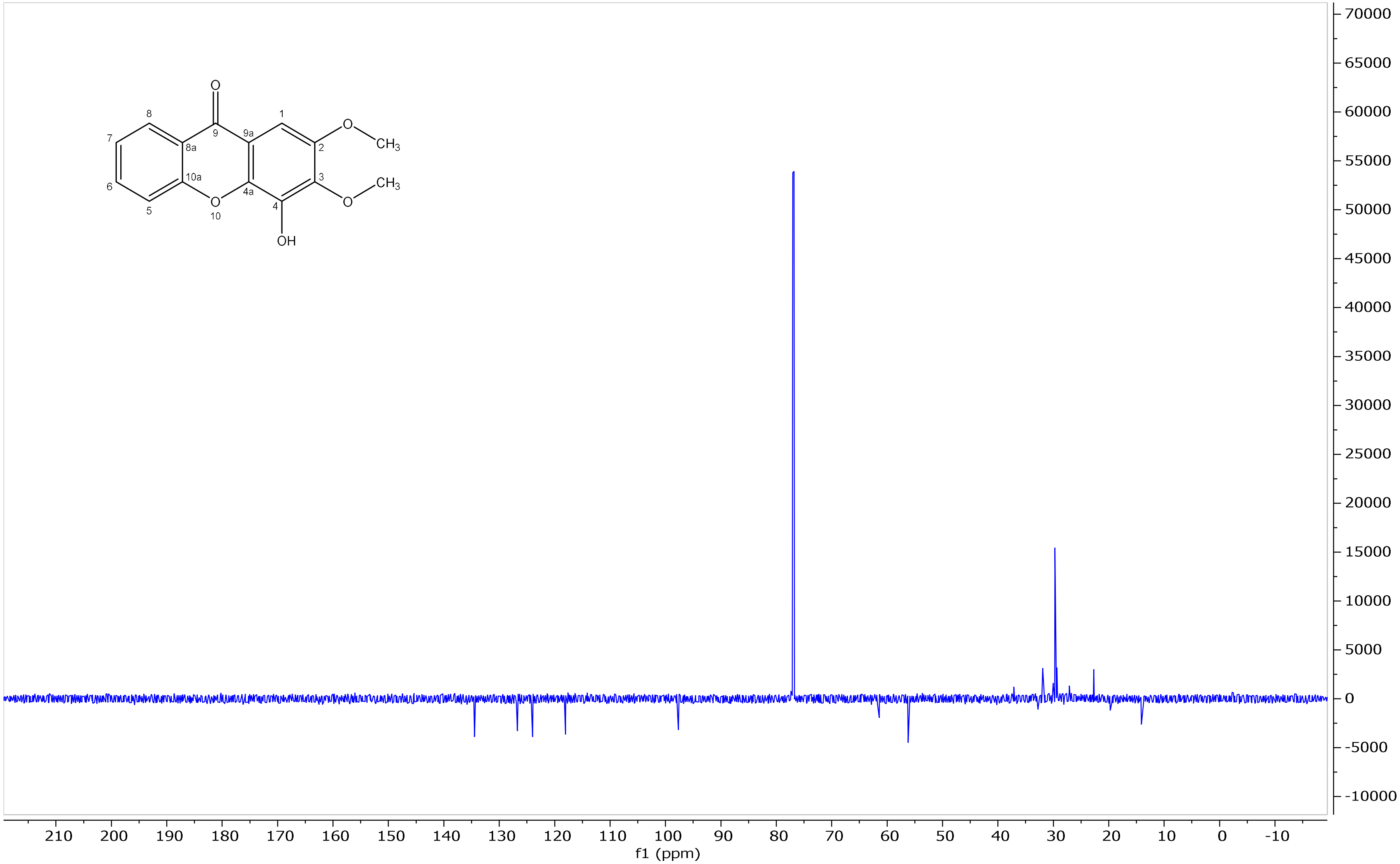

Plate S6 2D COSY NMR spectrum of Compound **1** ($CDCl_3$)

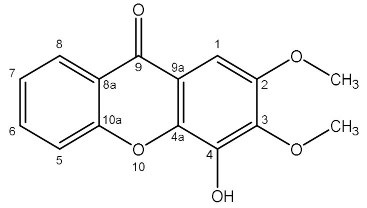

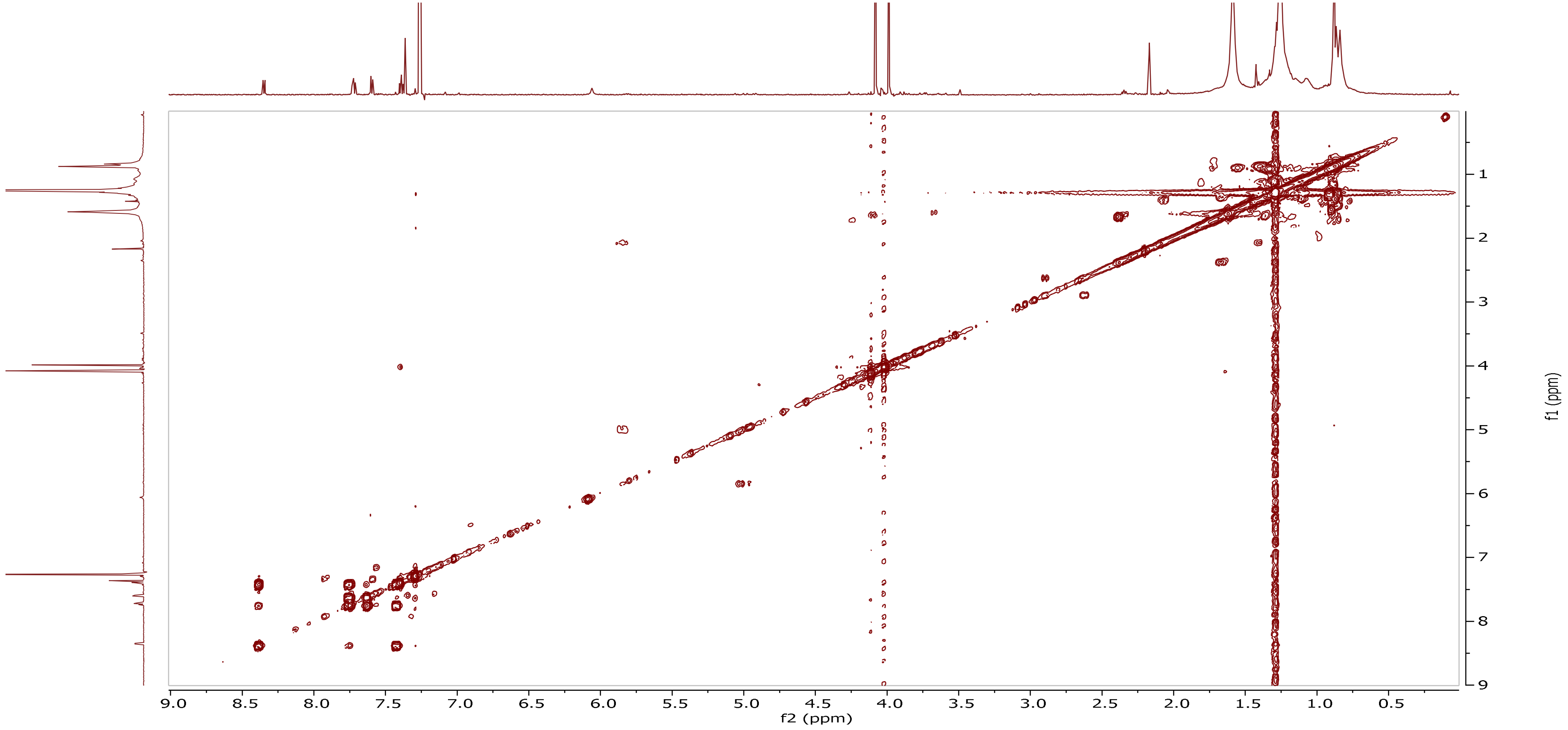

Plate S7 2D HSQC spectrum of Compound **1** ($CDCl_3$)

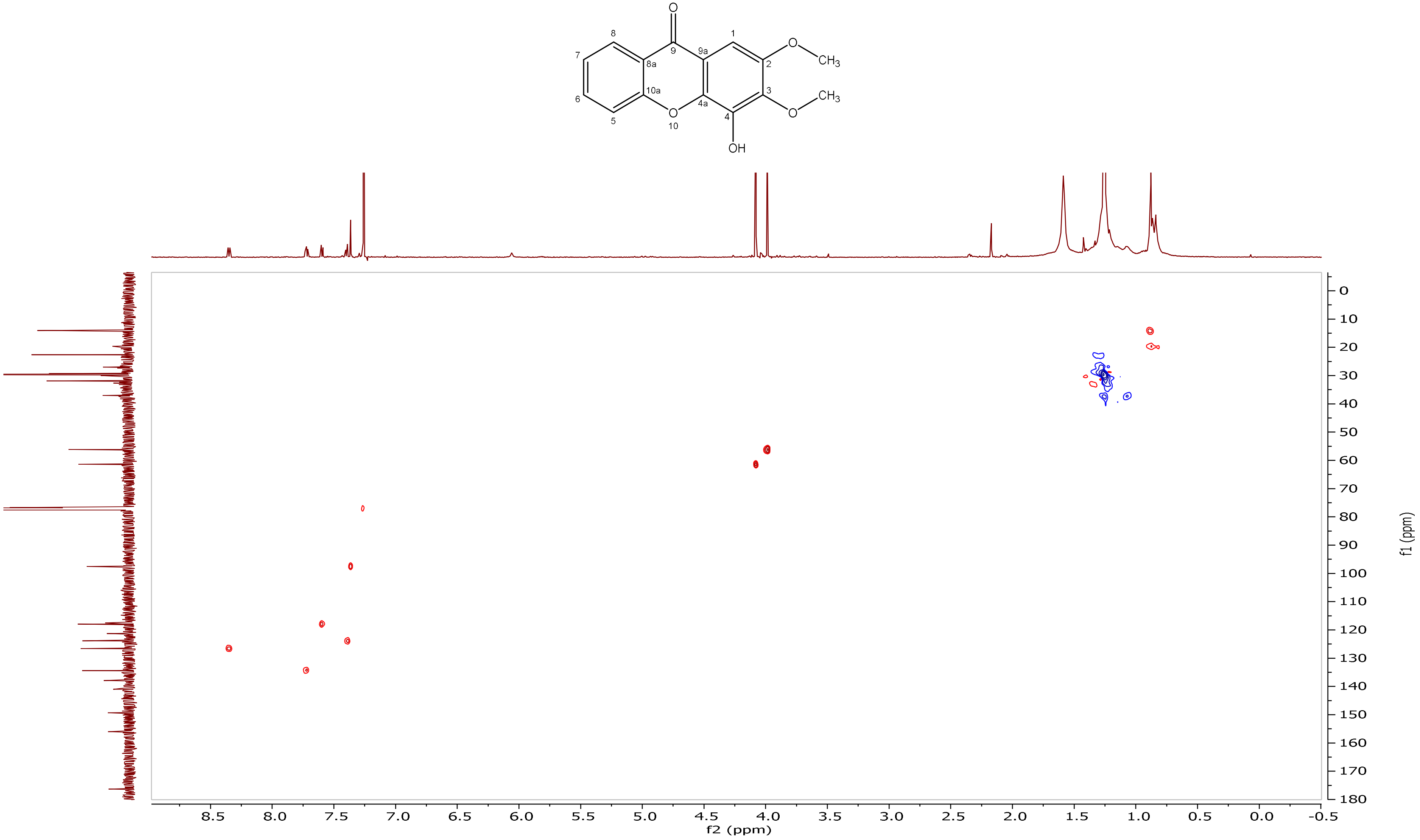

Plate S8 2D HMBC spectrum of Compound **1** ($CDCl_3$)

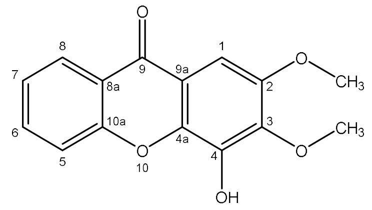

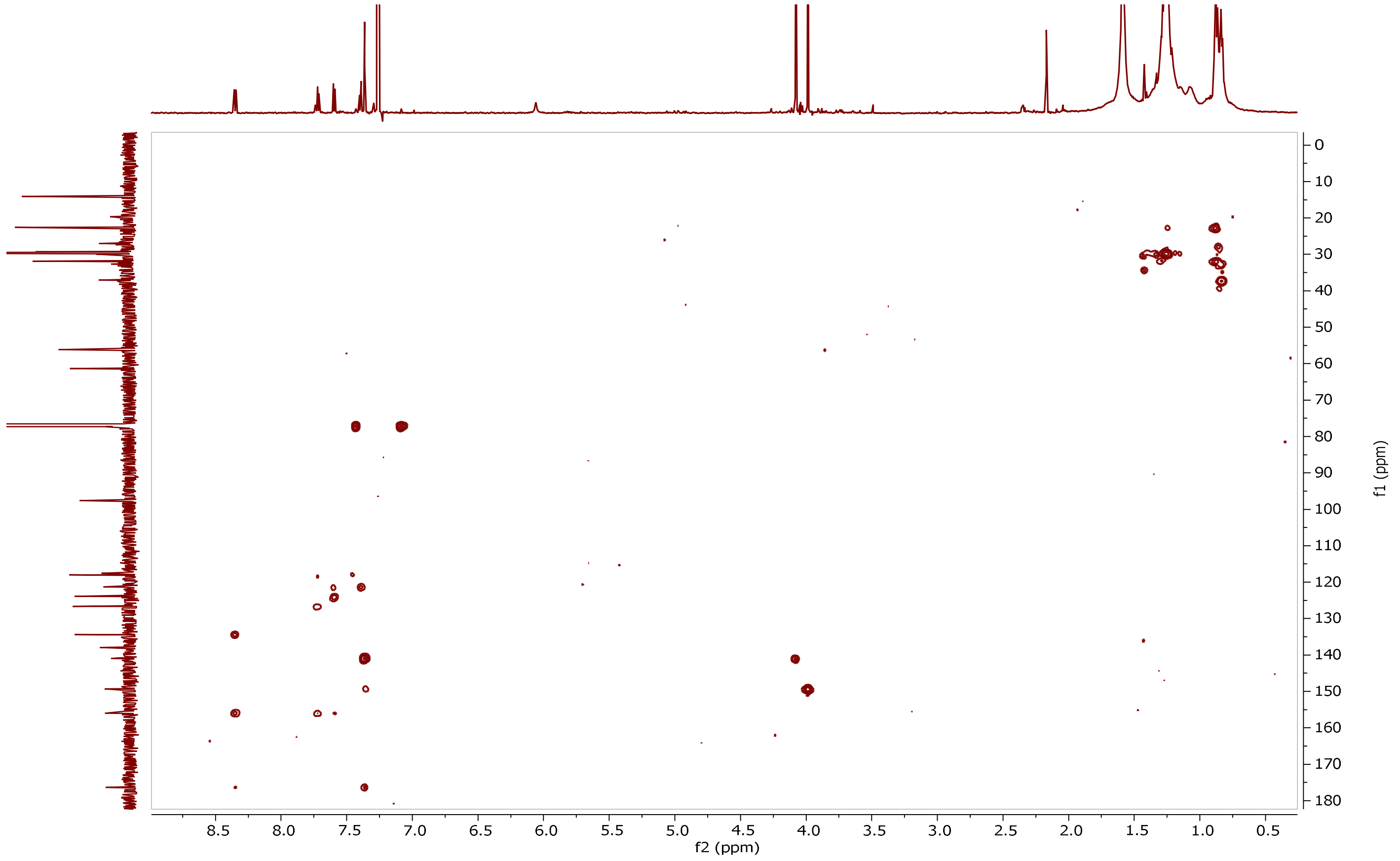

Plate S9 HR-ESI MS spectrum of Compound 2

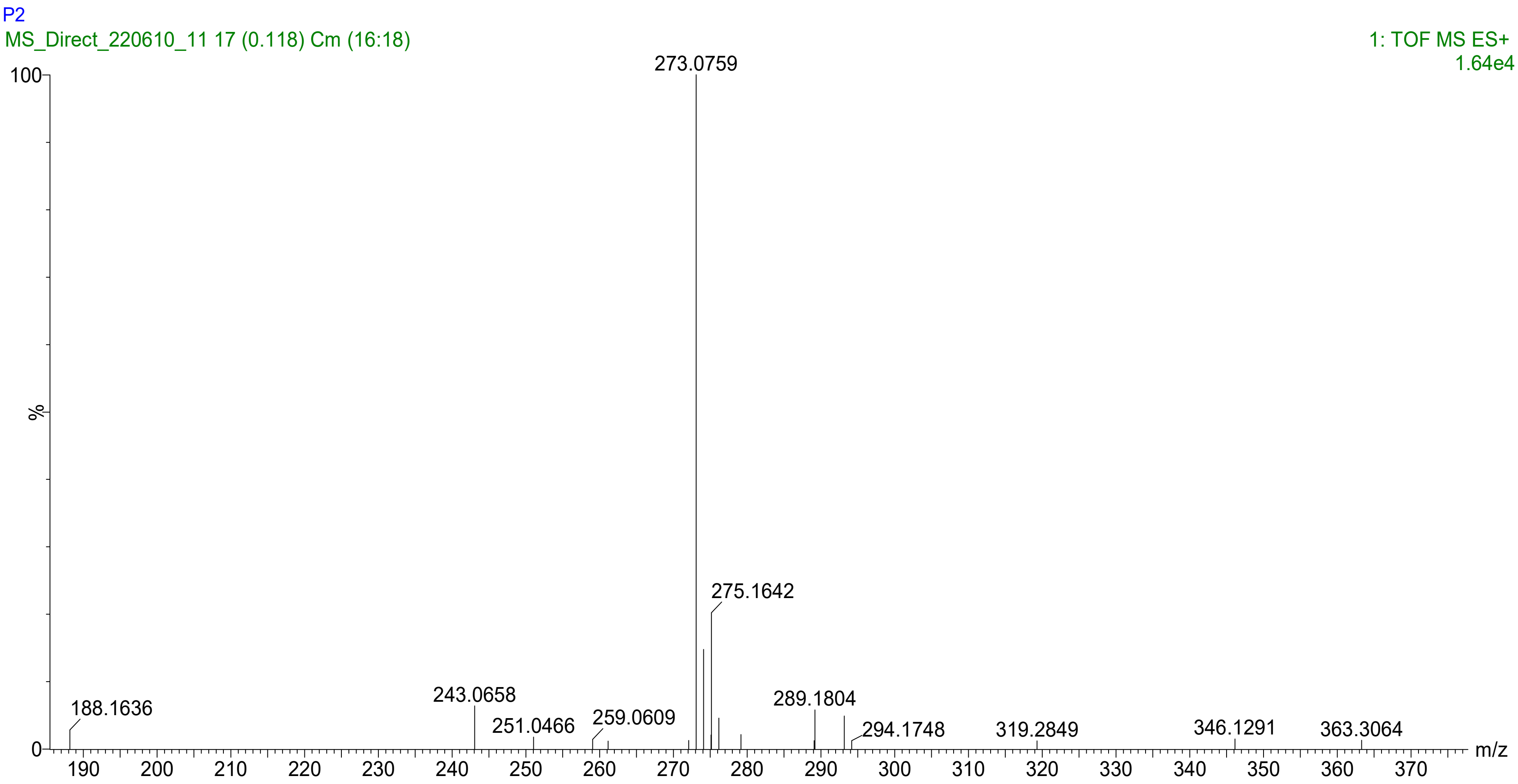

Plate S10 IR spectrum of Compound **2**.

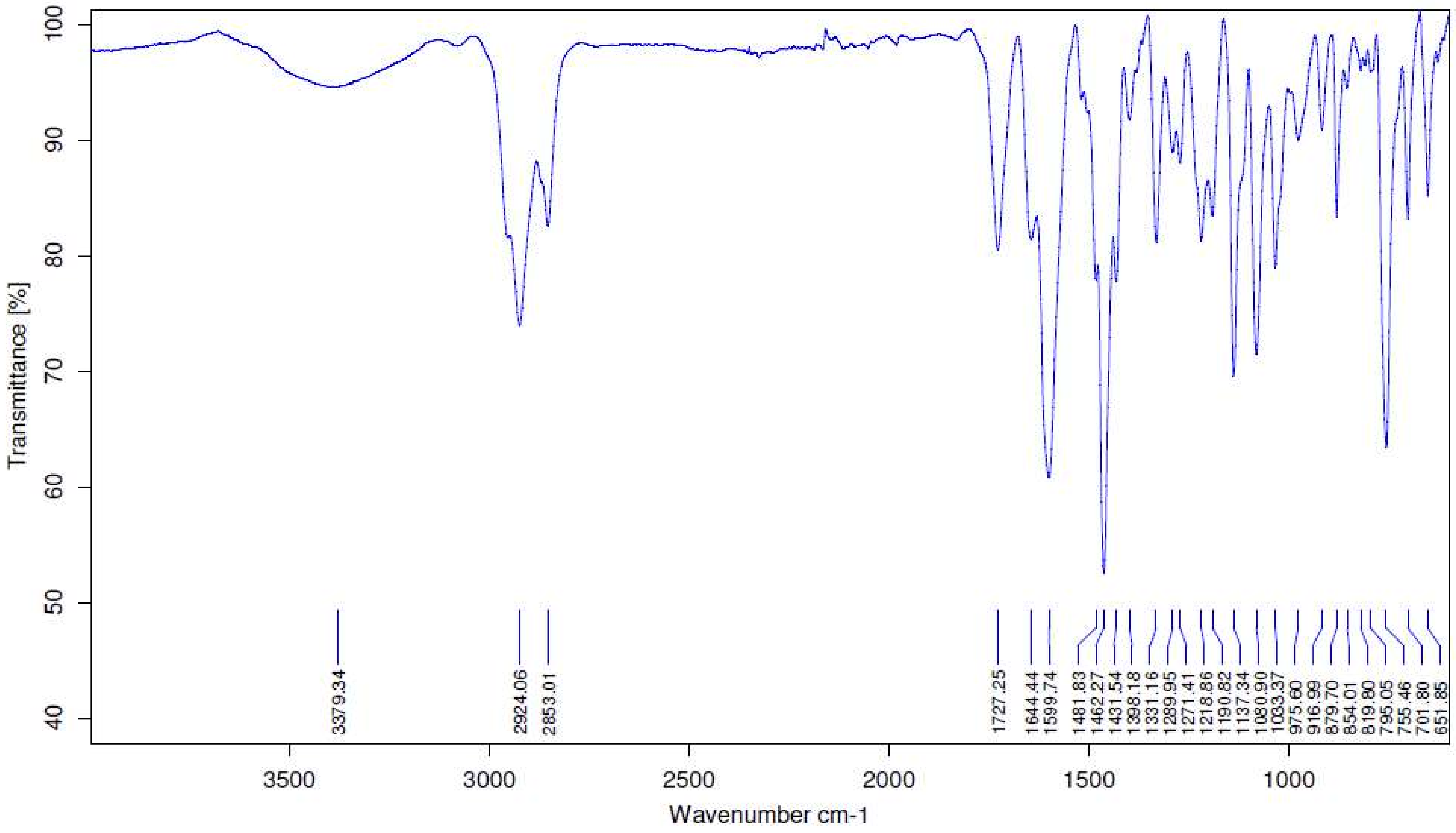

Plate S11 ^{1}H NMR (600 MHz) spectrum of Compound **2** $CDCl_3$

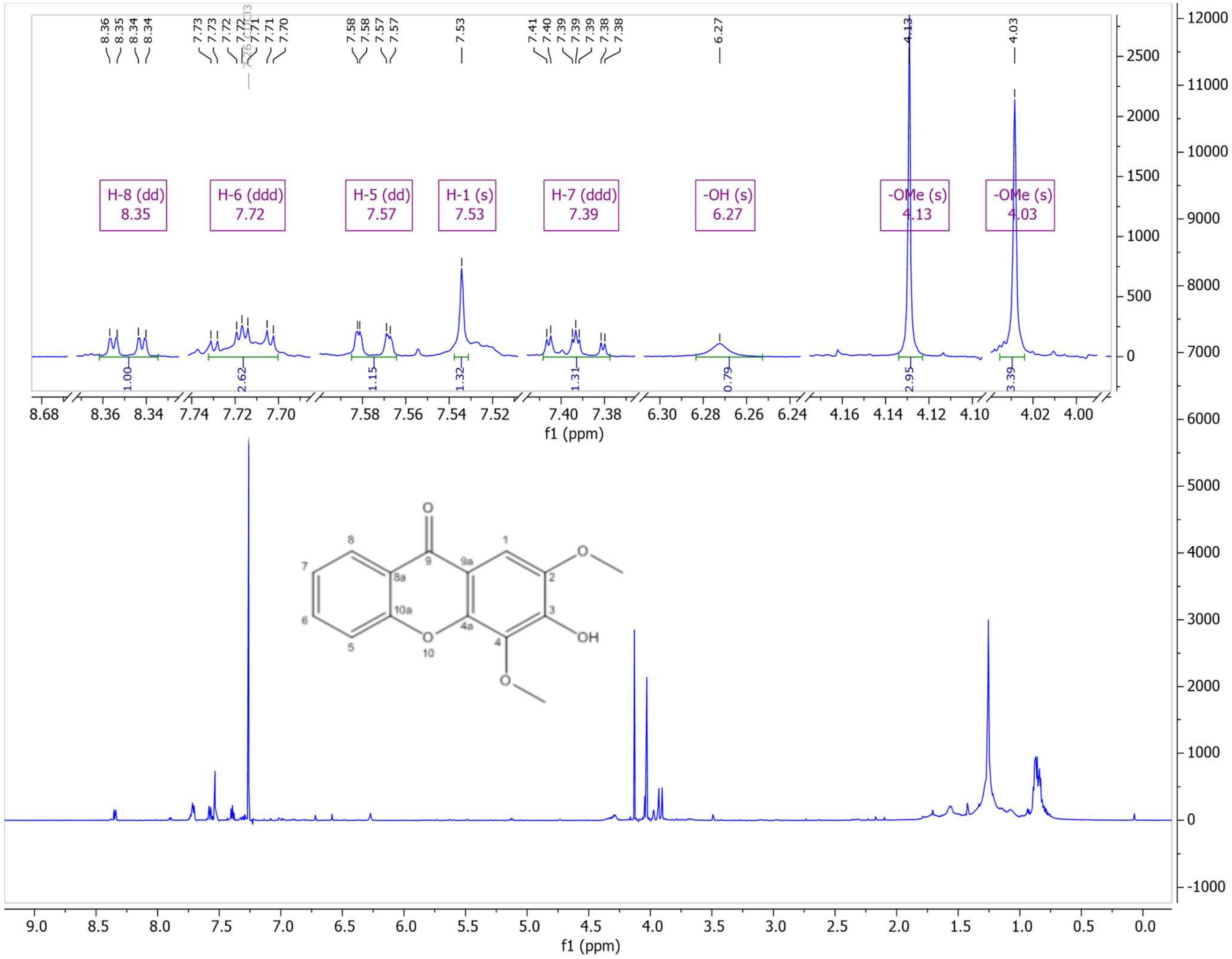

Plate S12 ^{13}C NMR (150 MHz) spectrum of Compound 2 $CDCl_3$

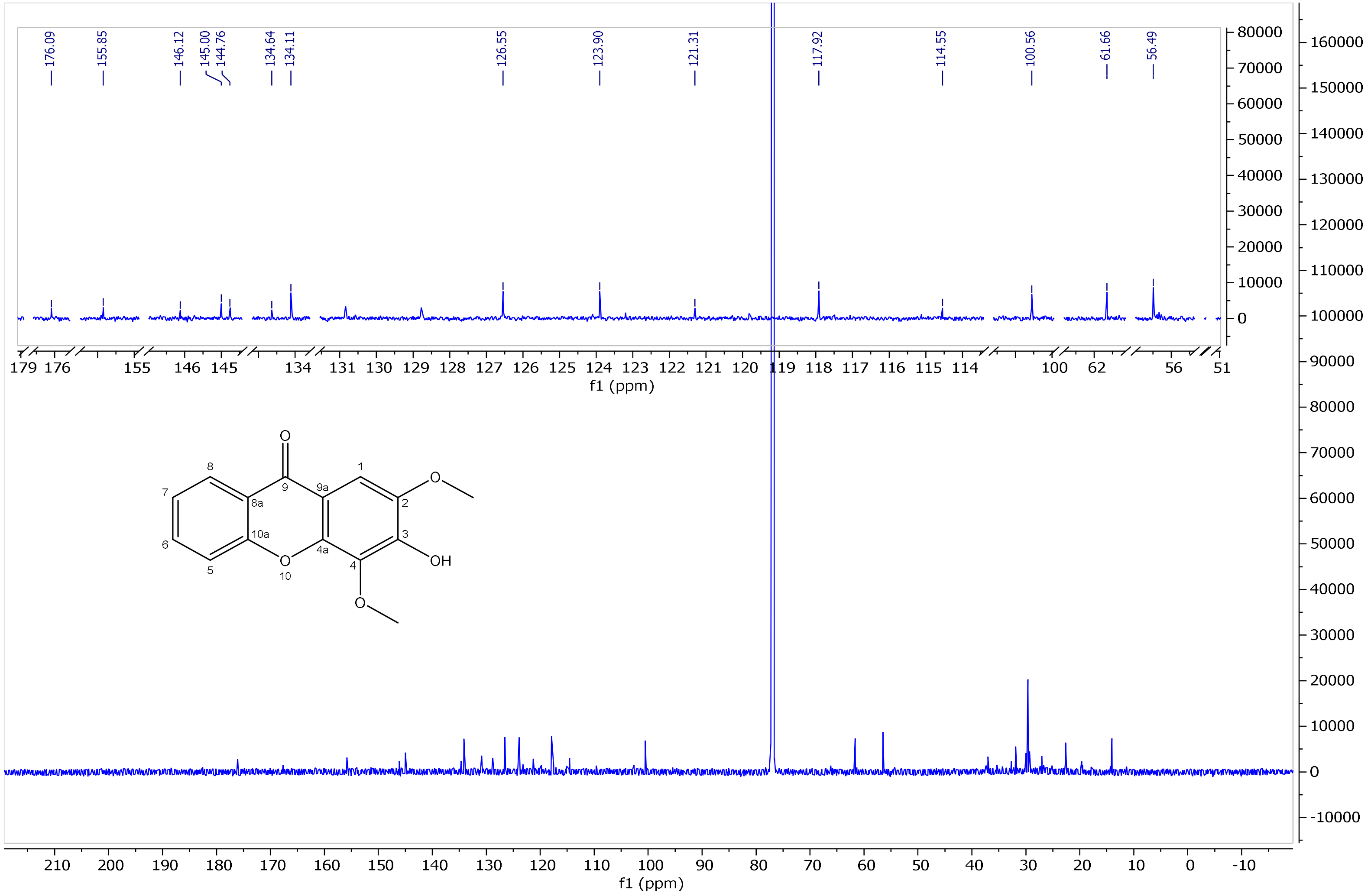

Plate S13 ^{13}C APT NMR (150 MHz) spectrum of Compound 2 $CDCl_3$

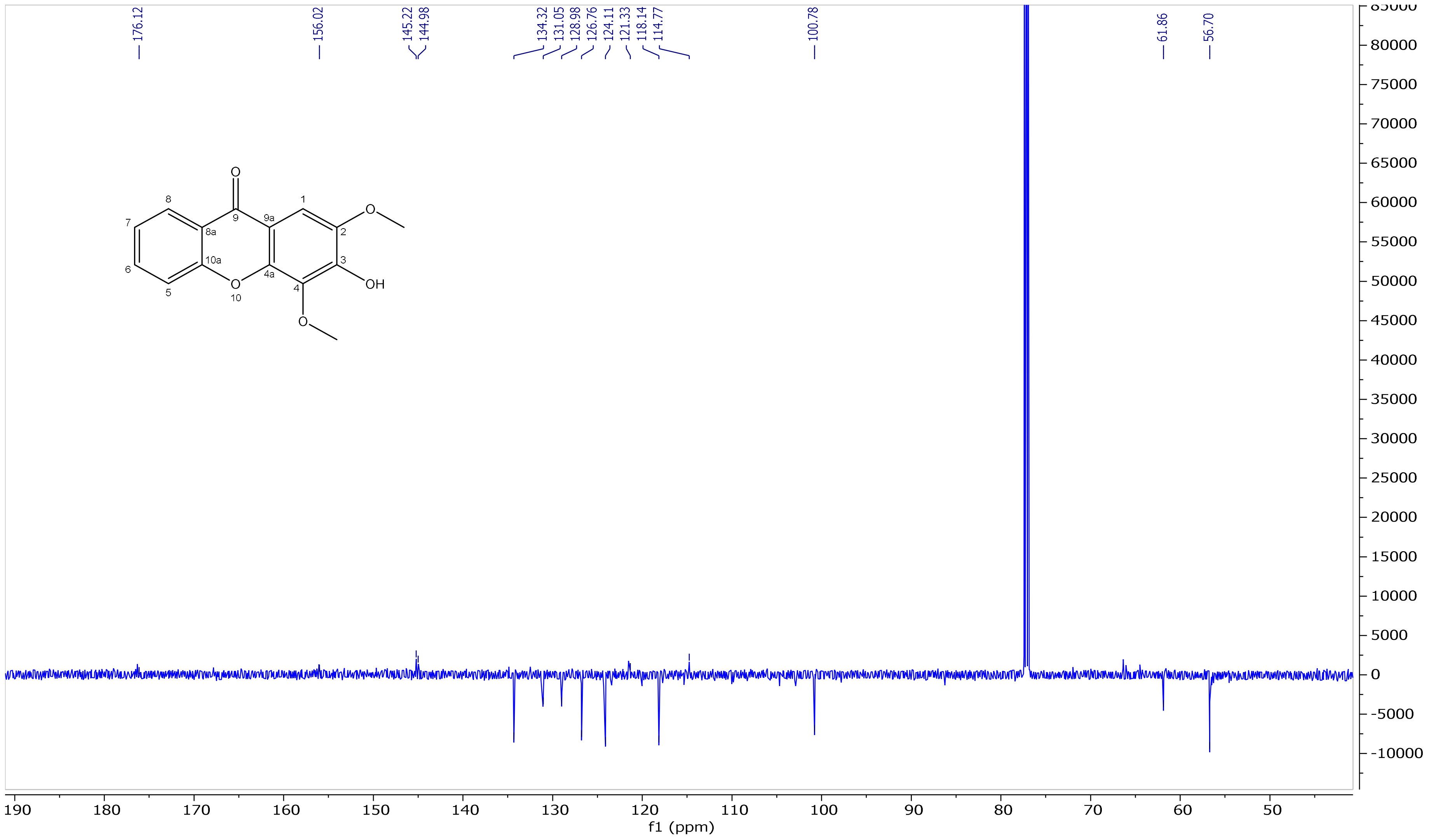

Plate S14 2D COSY NMR spectrum of Compound 2 CDCl₃

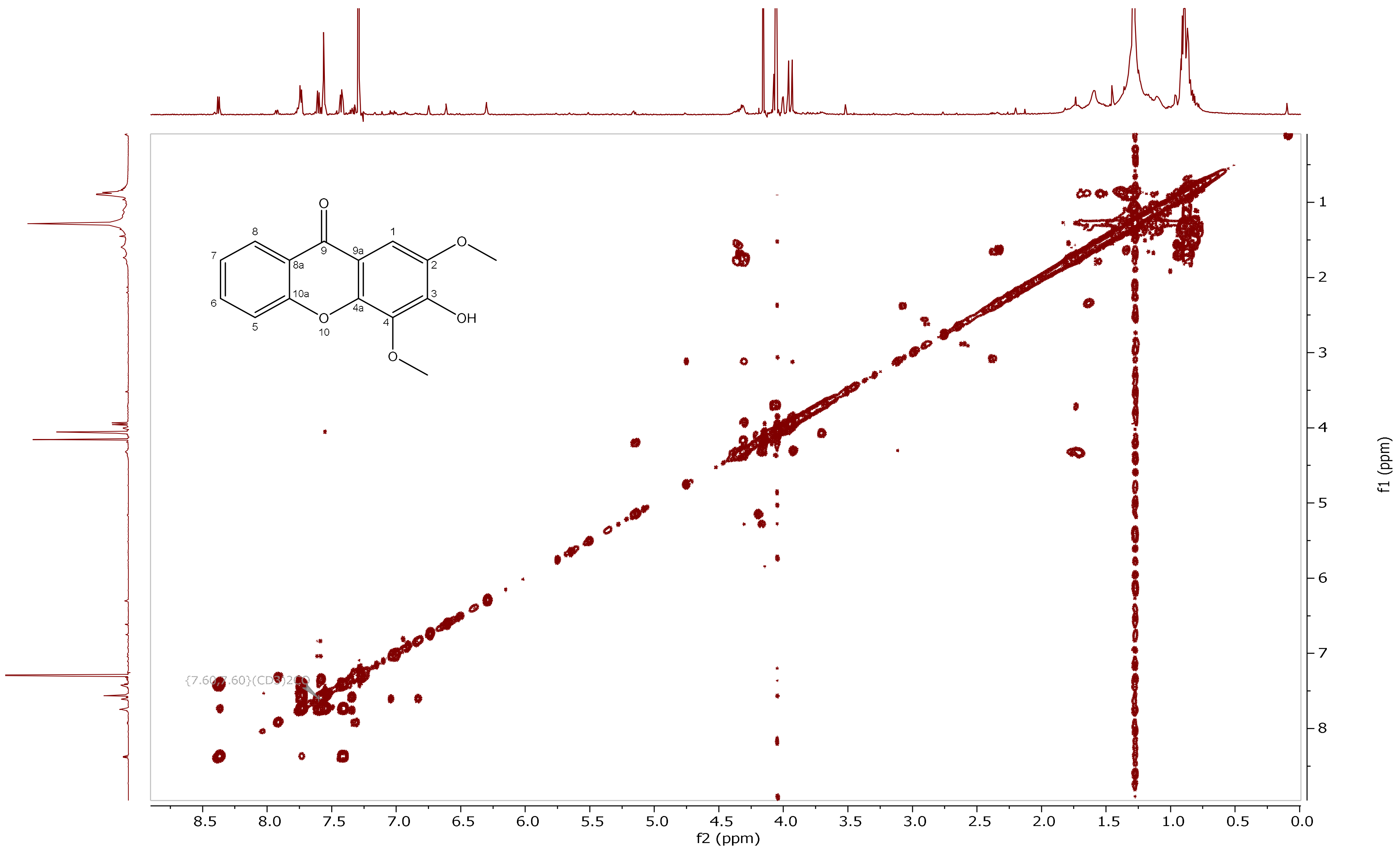

Plate S15 2D HSQC spectrum of Compound 2 CDCl3

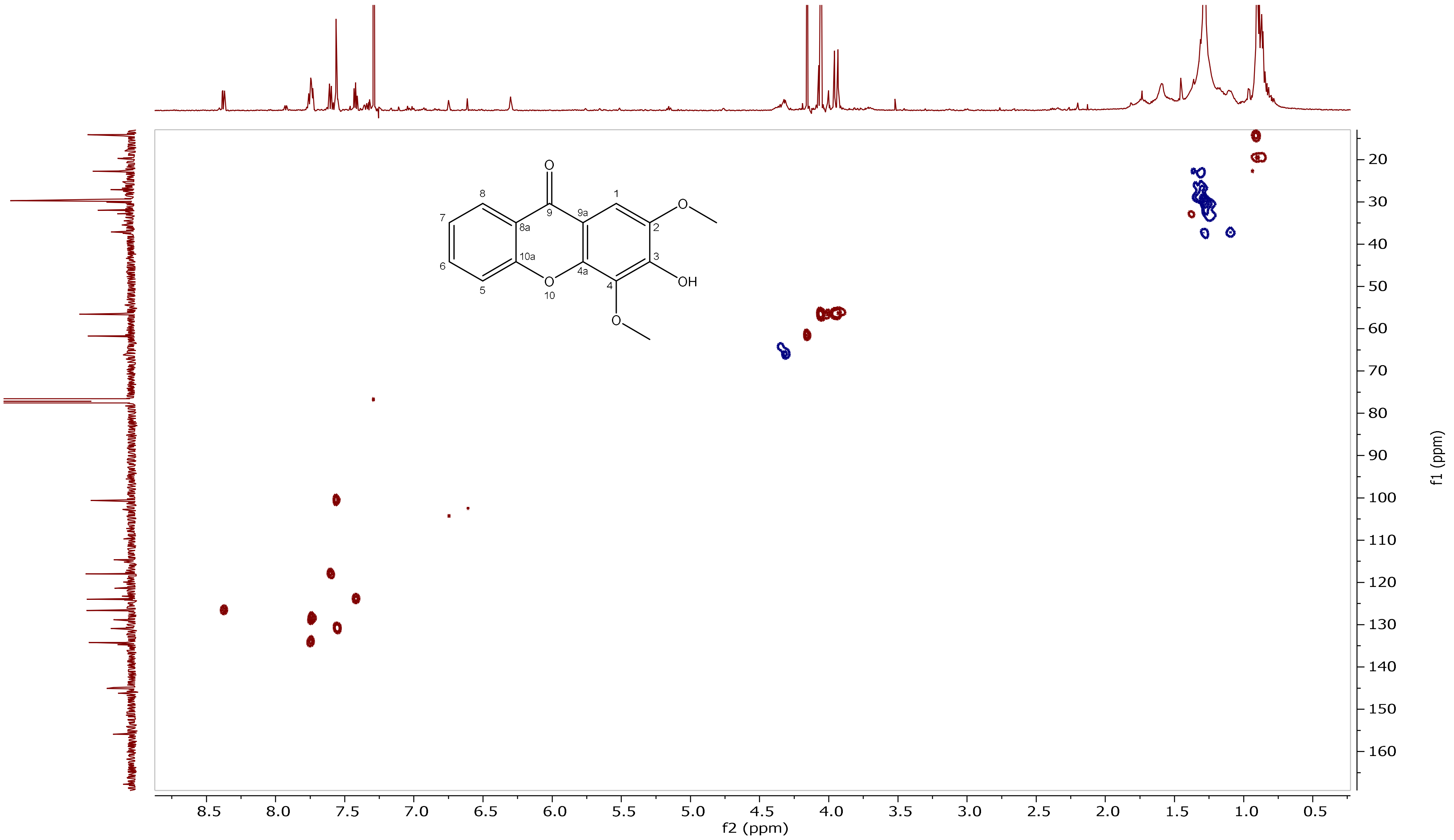

Plate S16 2D HMBC spectrum of Compound 2 $CDCl_3$

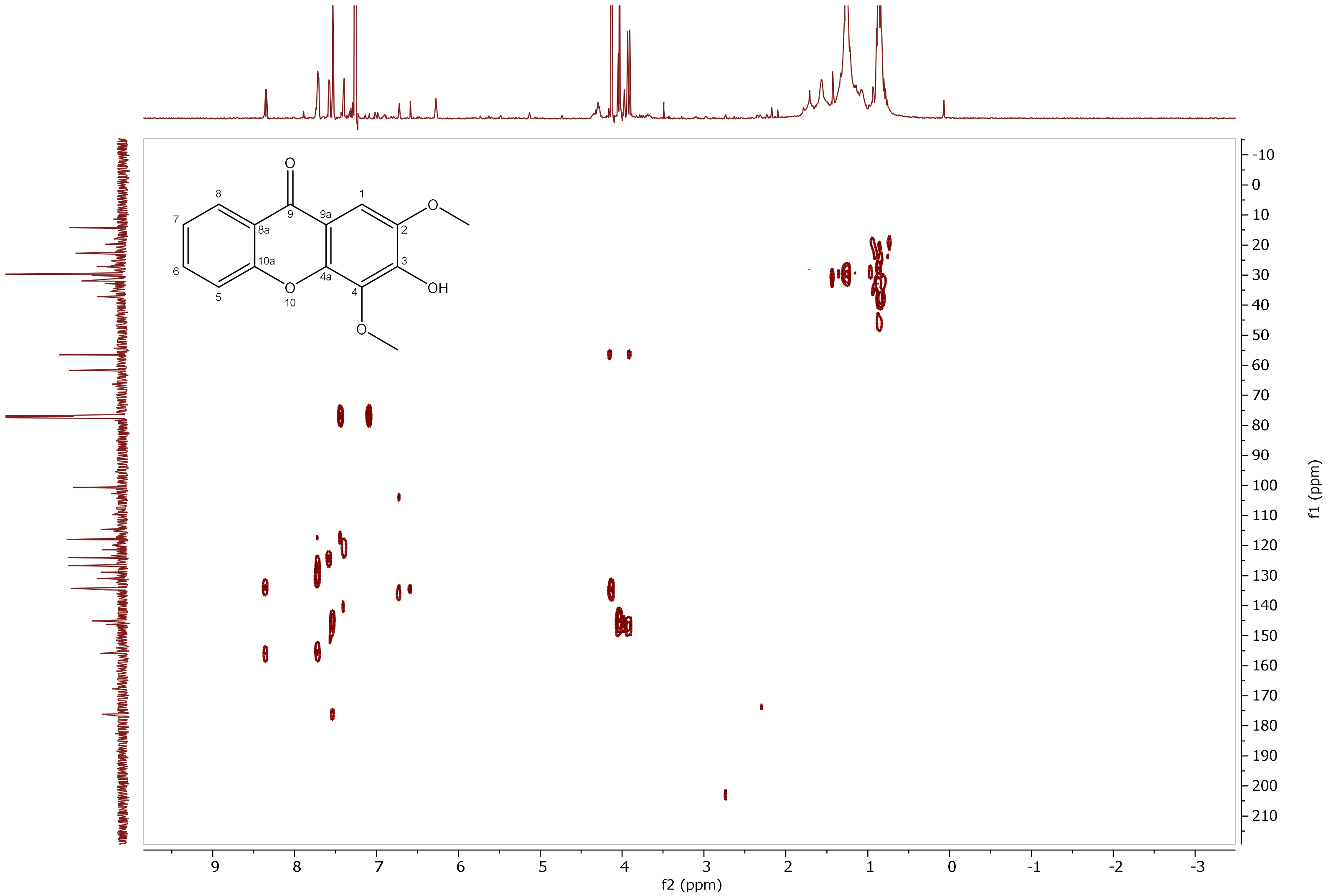

Plate S17 HR-ESI MS of Compound 3

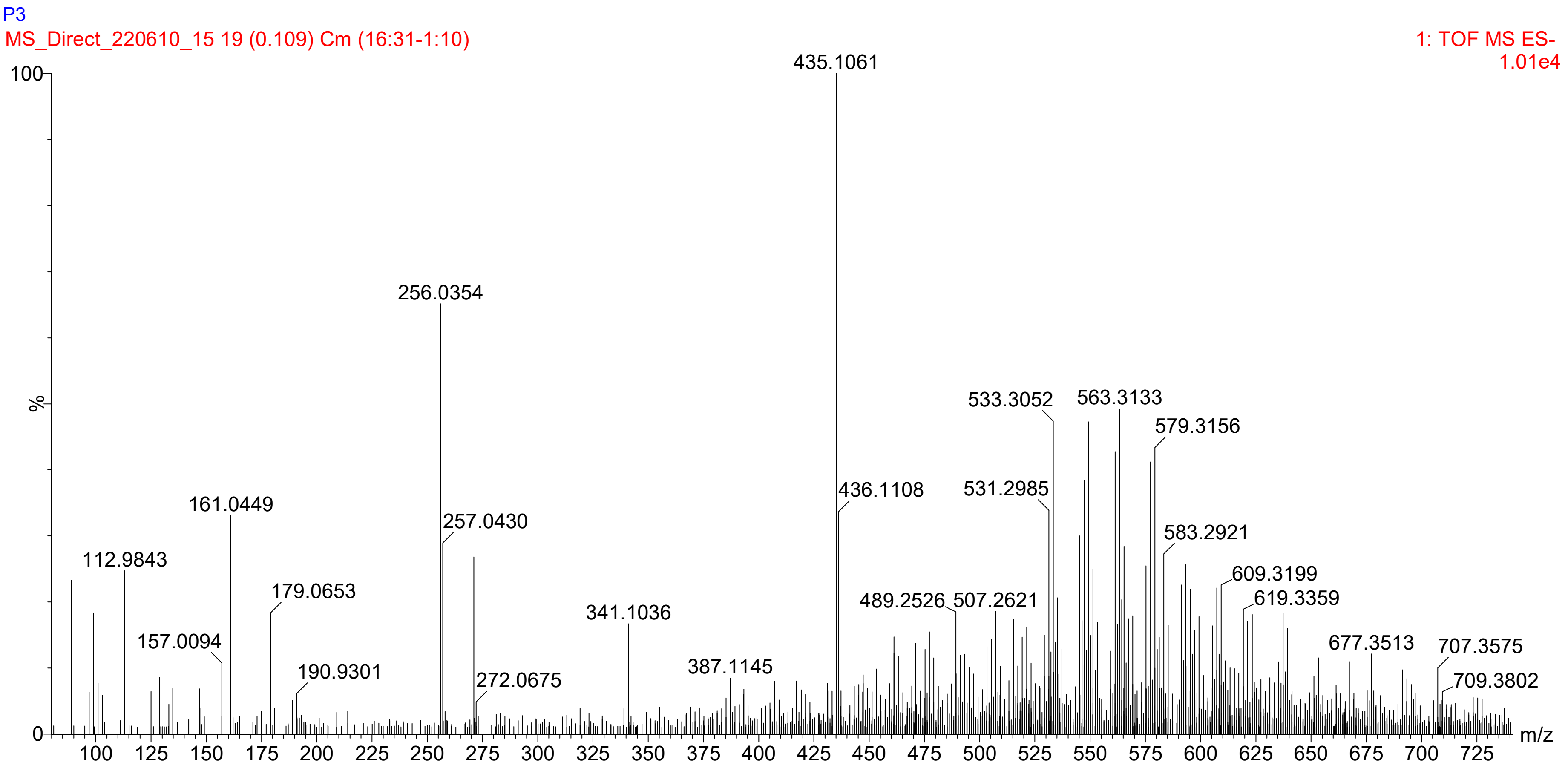

Plate S18 IR spectrum of Compound 3

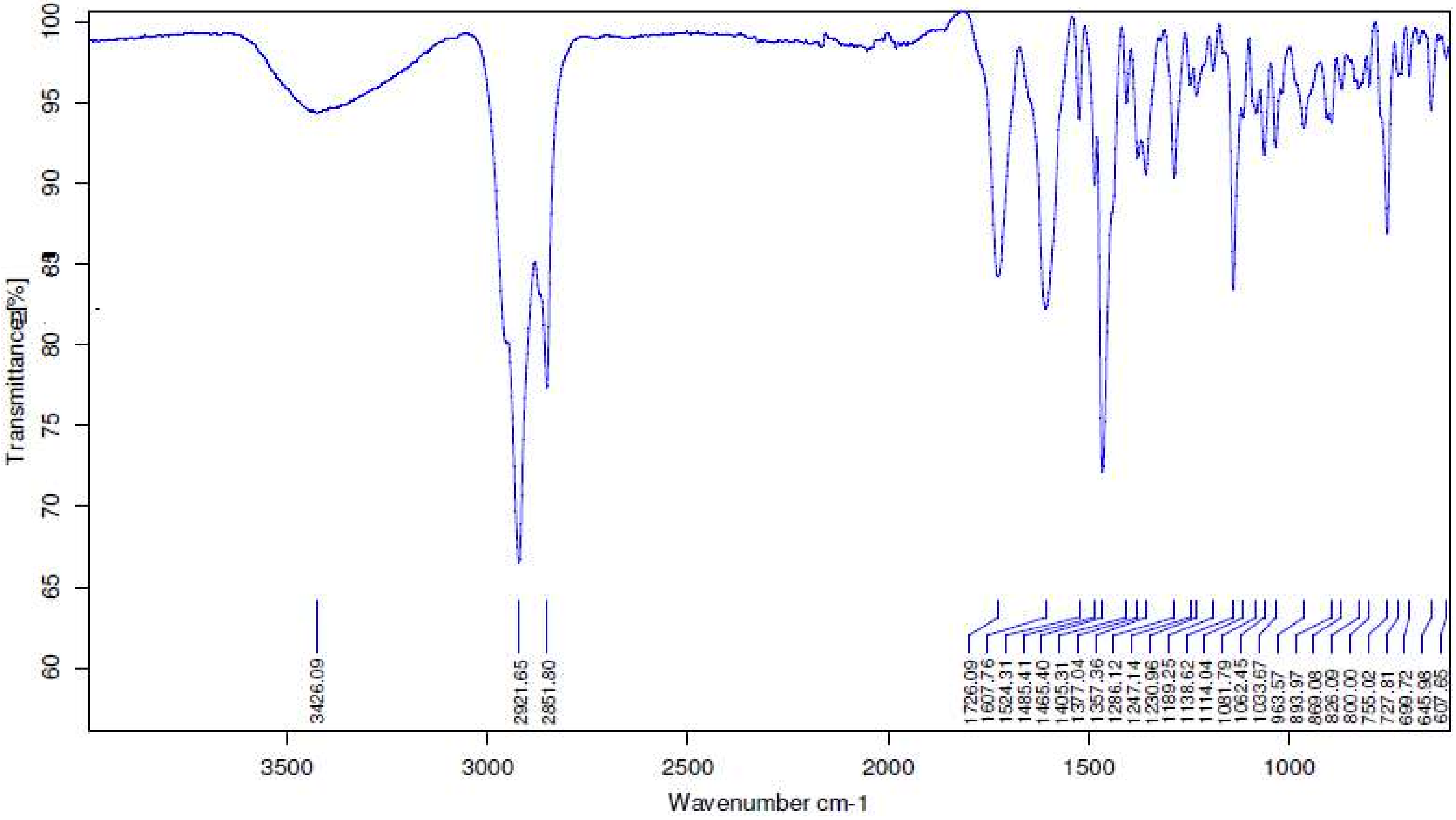

Plate S19 ¹H NMR (600 MHz) spectrum of Compound 3 $CDCl_3$

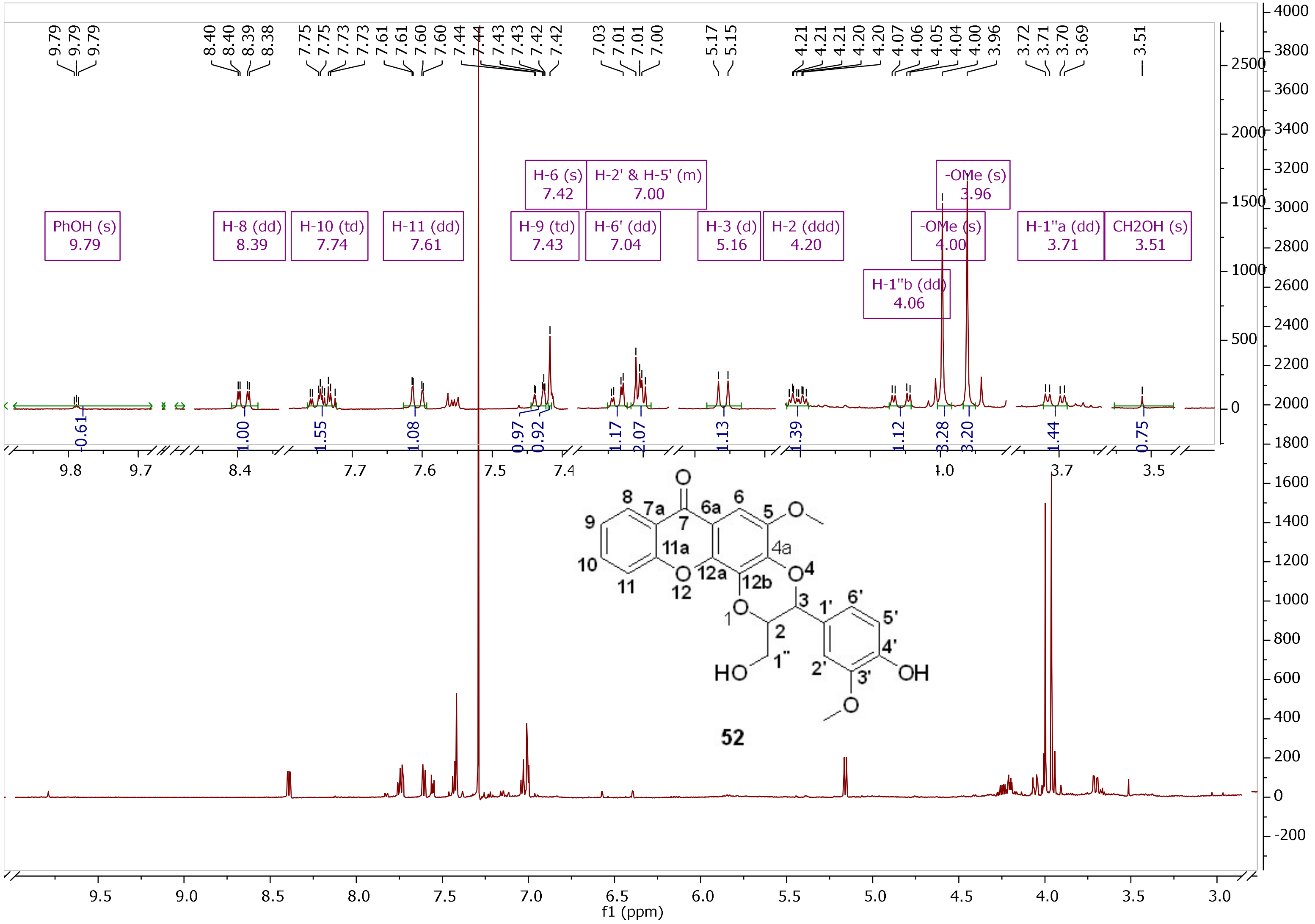

Plate S20 ^{13}C NMR (150 MHz) spectrum of Compound **3** $CDCl_3$

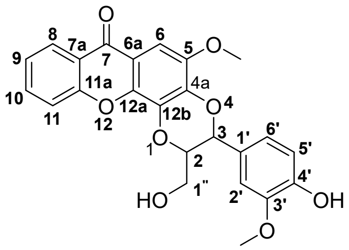

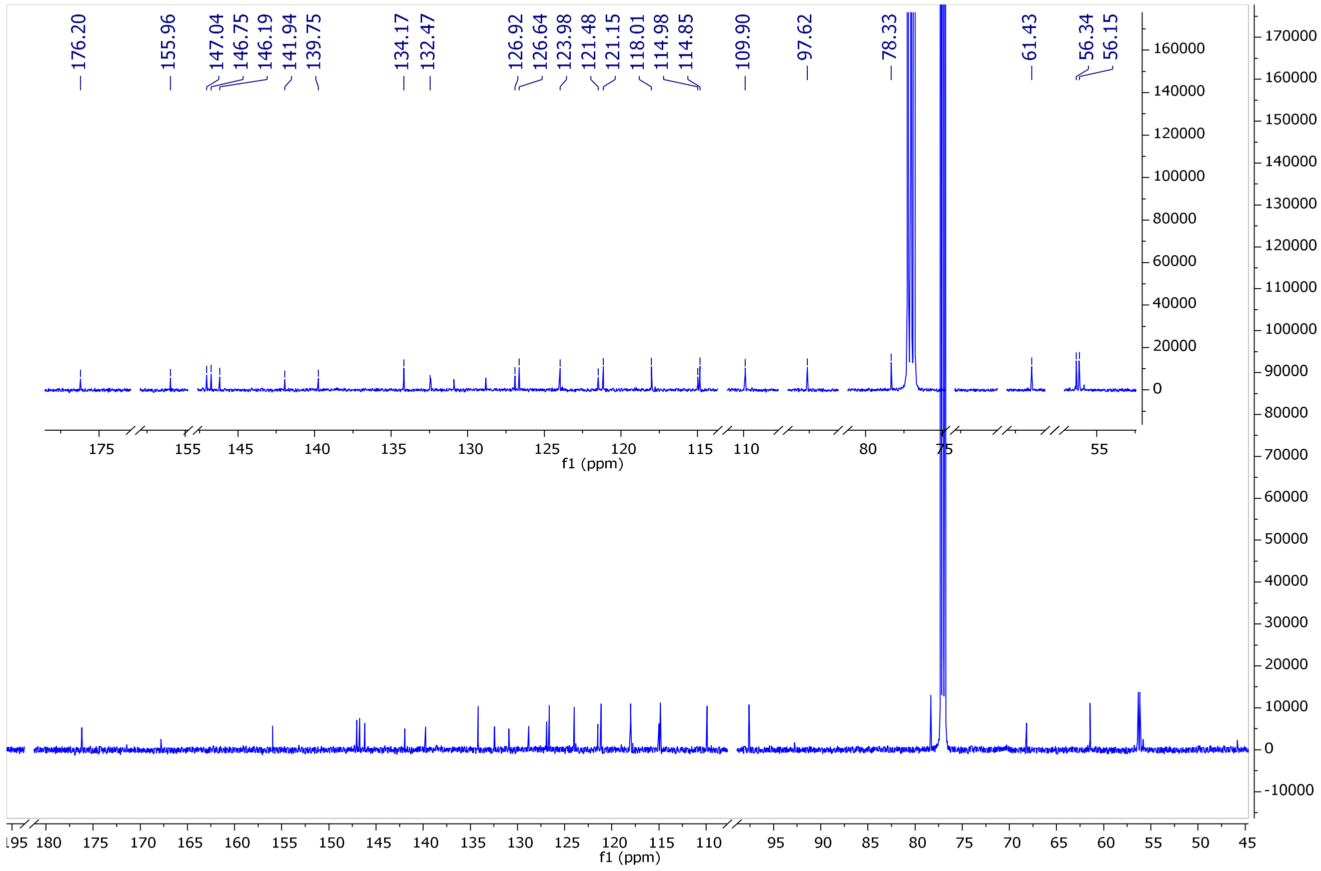

Plate S21 ^{13}C APT NMR (150 MHz) spectrum of Compound 3 $CDCl_3$

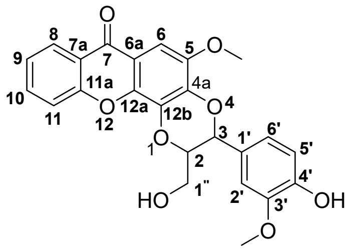

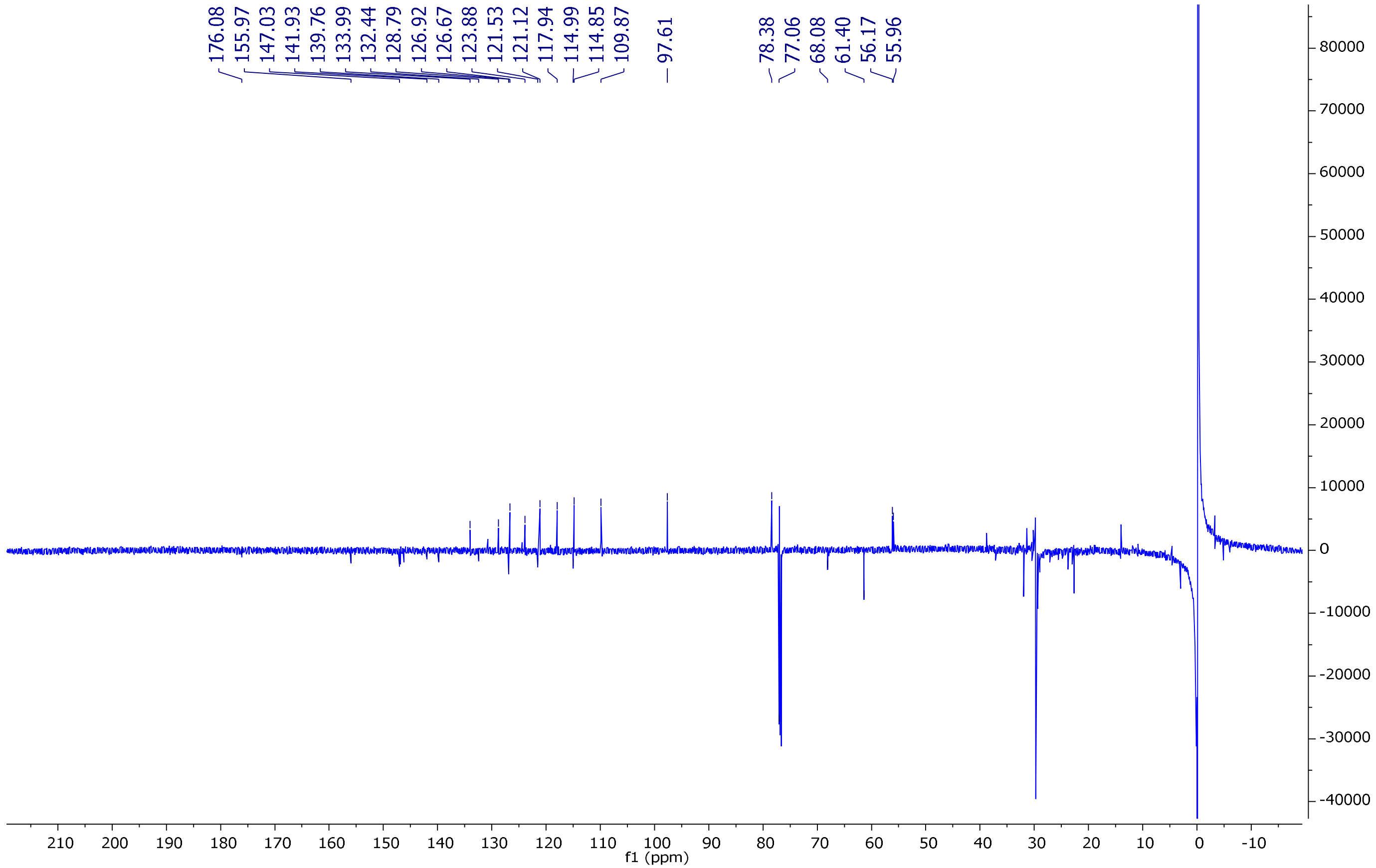

Plate S22 2D COSY NMR spectrum of Compound 3 CDCl₃

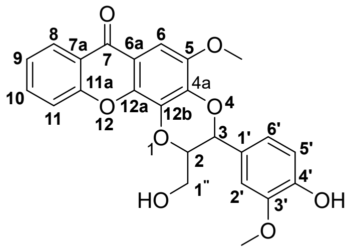

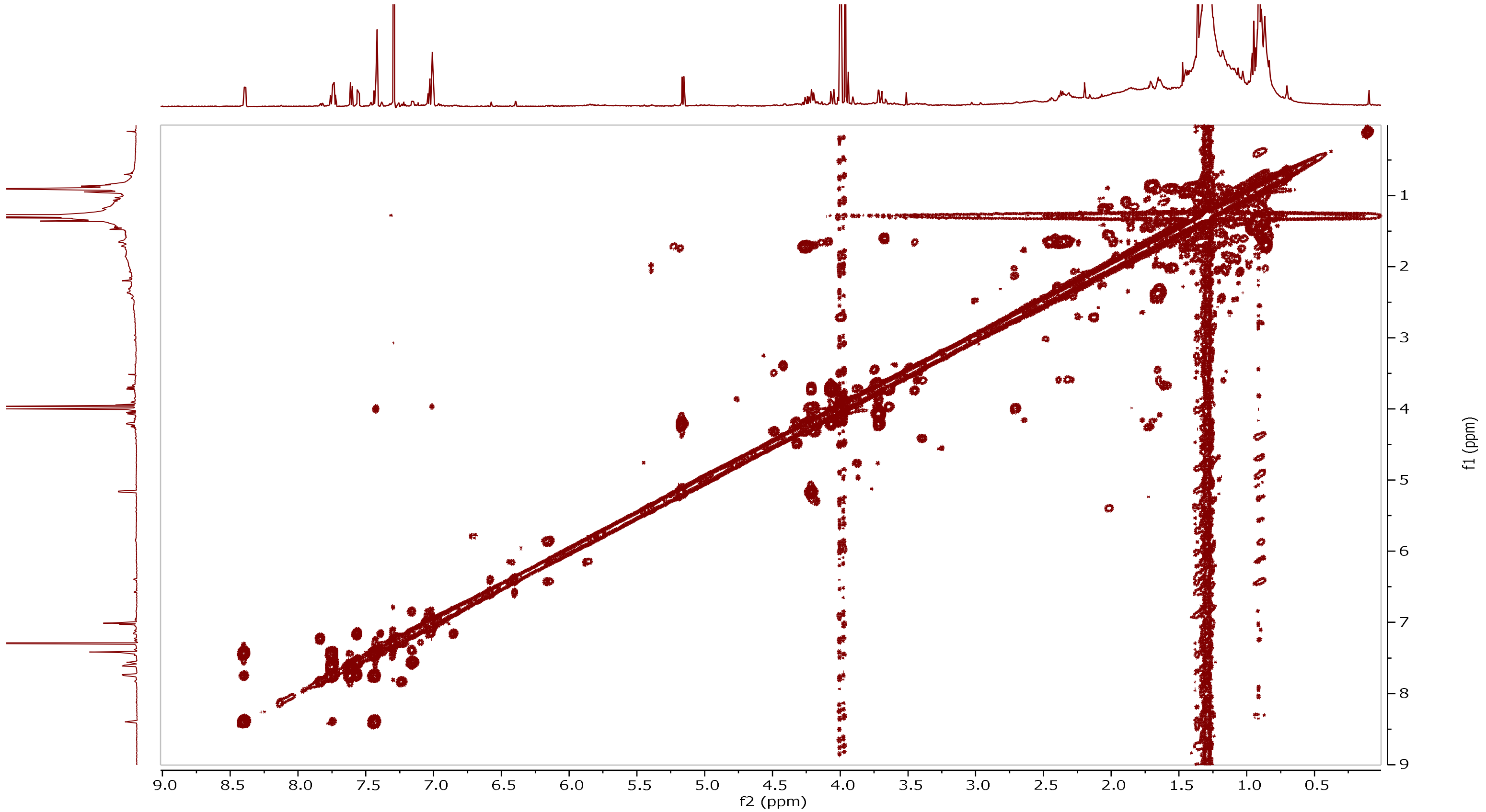

Plate S23 2D HSQC spectrum of Compound **3** $CDCl_3$

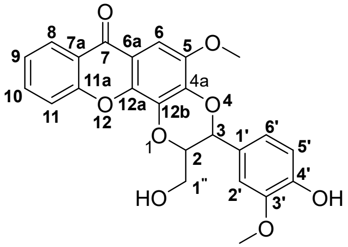

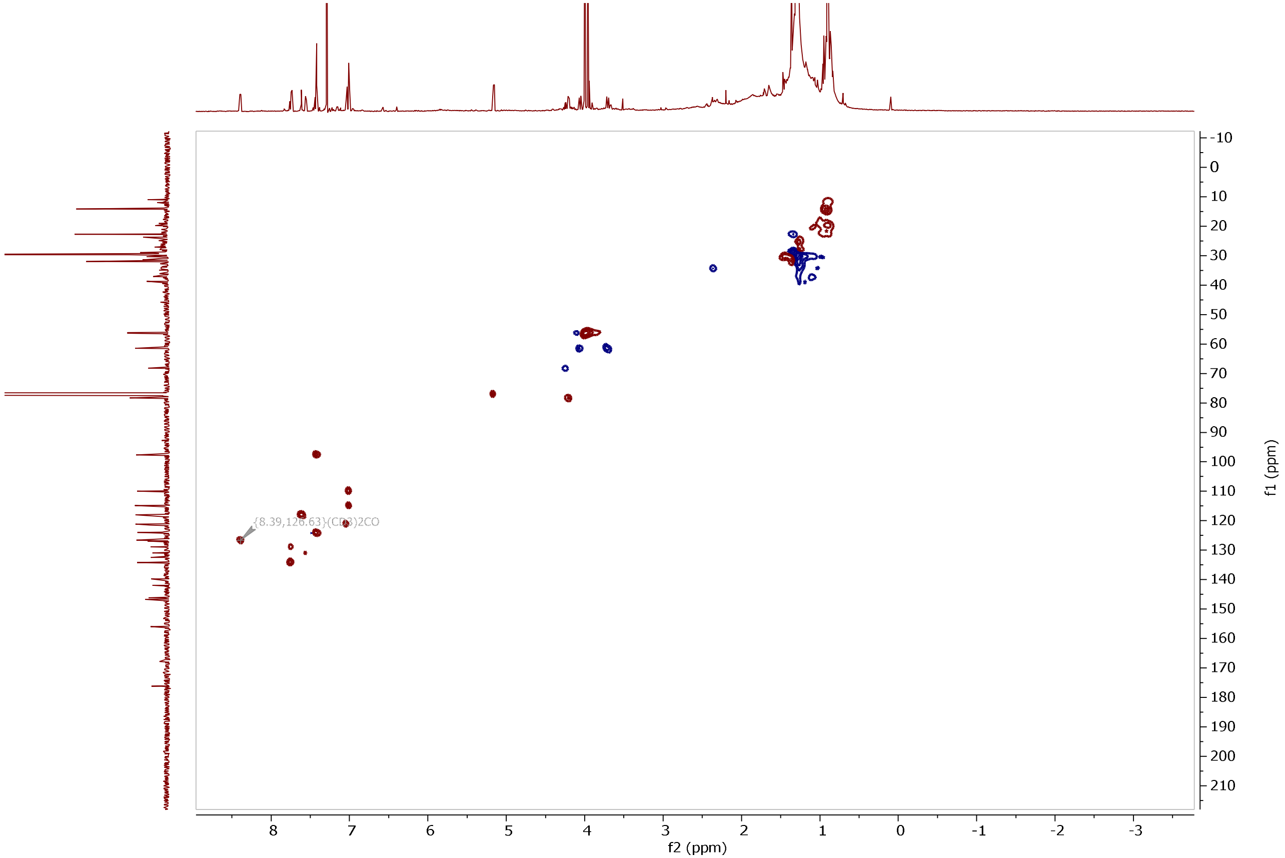

Plate S24 2D HMBC spectrum of Compound **3** $CDCl_3$

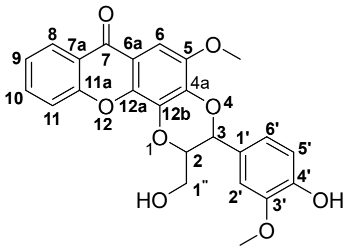

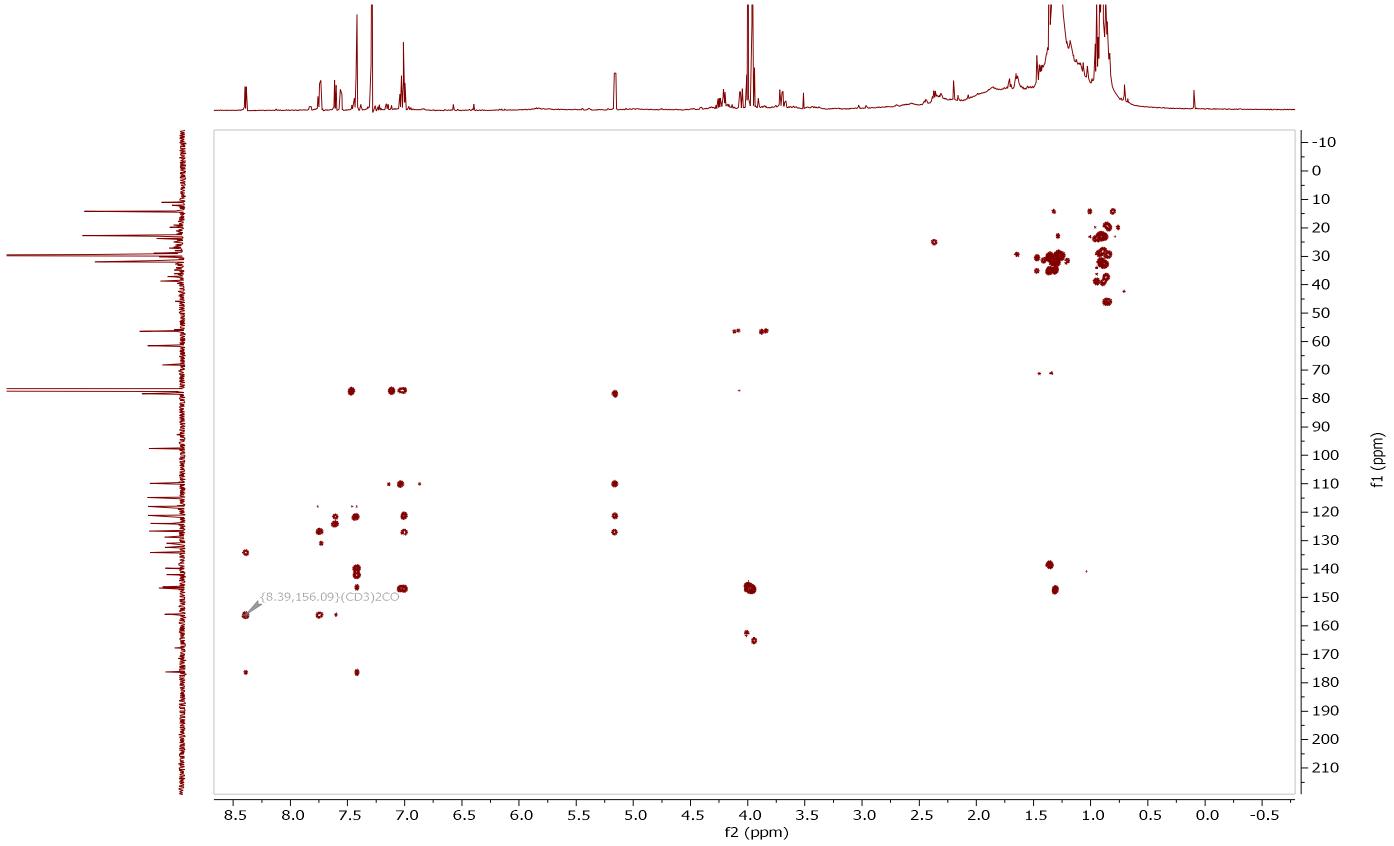

Plate S25 2D NOESY spectrum of Compound **3** $CDCl_3$

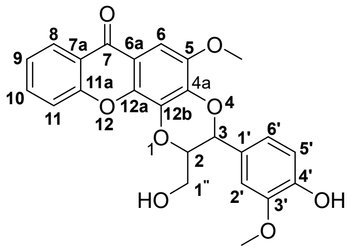

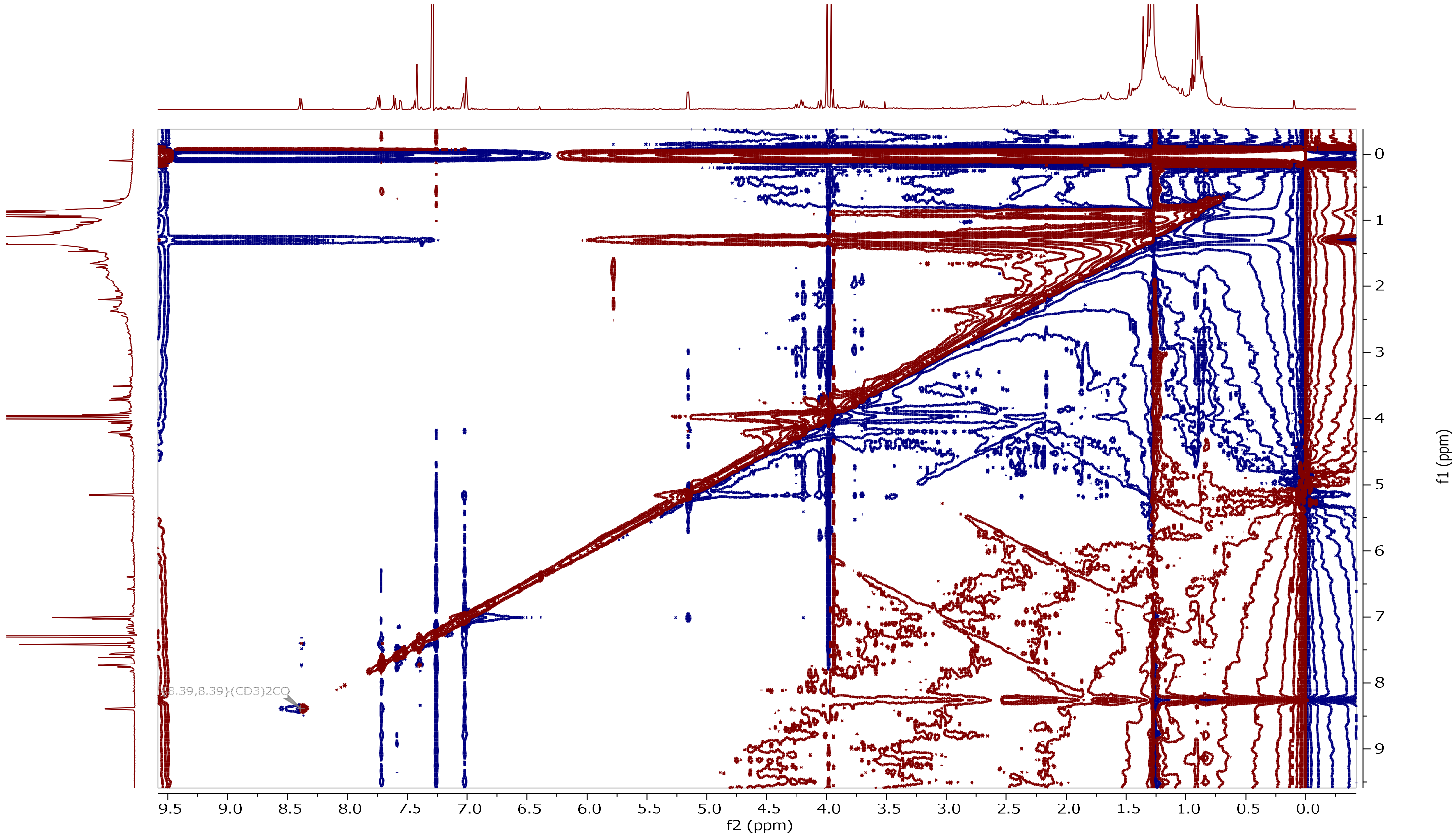

Supplement: Supplementary file 1 [file molecules-30-03530-s001.zip › molecules-3771876-supplementary.pdf]
